# Face Mask Use in the Community for Reducing the Spread of COVID-19: A Systematic Review

**DOI:** 10.3389/fmed.2020.594269

**Published:** 2021-01-12

**Authors:** Daniela Coclite, Antonello Napoletano, Silvia Gianola, Andrea del Monaco, Daniela D'Angelo, Alice Fauci, Laura Iacorossi, Roberto Latina, Giuseppe La Torre, Claudio M. Mastroianni, Cristina Renzi, Greta Castellini, Primiano Iannone

**Affiliations:** ^1^Centro Eccellenza Clinica, Qualità e Sicurezza delle Cure, Istituto Superiore di Sanità, Rome, Italy; ^2^Unit of Clinical Epidemiology, IRCCS Istituto Ortopedico Galeazzi, Milan, Italy; ^3^Directorate General for Economics, Statistics and Research, Bank of Italy, Rome, Italy; ^4^Department of Public Health and Infectious Diseases, Sapienza University of Rome, Rome, Italy; ^5^Institute of Epidemiology & Health Care, University College London-UCL, London, United Kingdom

**Keywords:** pandemics, face mask, prevention and control [MeSH], SARS—CoV−2, COVID−19, disease outbreaks, community, systematic review

## Abstract

**Background:** Evidence is needed on the effectiveness of wearing face masks in the community to prevent SARS-CoV-2 transmission.

**Methods:** Systematic review and meta-analysis to investigate the efficacy and effectiveness of face mask use in a community setting and to predict the effectiveness of wearing a mask. We searched MEDLINE, EMBASE, SCISEARCH, The Cochrane Library, and pre-prints from inception to 22 April 2020 without restriction by language. We rated the certainty of evidence according to Cochrane and GRADE approach.

**Findings:** Our search identified 35 studies, including three randomized controlled trials (RCTs) (4,017 patients), 10 comparative studies (18,984 patients), 13 predictive models, nine laboratory experimental studies. For reducing infection rates, the estimates of cluster-RCTs were in favor of wearing face masks vs. no mask, but not at statistically significant levels (adjusted OR 0.90, 95% CI 0.78–1.05). Similar findings were reported in observational studies. Mathematical models indicated an important decrease in mortality when the population mask coverage is near-universal, regardless of mask efficacy. In the best-case scenario, when the mask efficacy is at 95%, the R0 can fall to 0.99 from an initial value of 16.90. Levels of mask filtration efficiency were heterogeneous, depending on the materials used (surgical mask: 45–97%). One laboratory study suggested a viral load reduction of 0.25 (95% CI 0.09–0.67) in favor of mask vs. no mask.

**Interpretation:** The findings of this systematic review and meta-analysis support the use of face masks in a community setting. Robust randomized trials on face mask effectiveness are needed to inform evidence-based policies.

**PROSPERO registration:** CRD42020184963.

## Introduction

Coronavirus disease 2019 (COVID-19) is a new, rapidly emerging infectious disease caused by a novel coronavirus, SARS-CoV-2 (Severe Acute Respiratory Syndrome CoronaVirus-2), which is primarily transmitted via droplets during close unprotected contact with an infector and fomites (1, 2). The virus is genetically similar to the coronaviruses that caused Severe Acute Respiratory Syndrome (SARS) and the Middle East respiratory syndrome (MERS), but SARS-CoV-2 appears to have greater transmissibility and lower pathogenicity than the aforementioned viruses (3). Preliminary estimates of the basic reproduction number (*R*_0_) of SARS-CoV-2, as a metric for transmissibility, range from 2.8 to 5.5, in the absence of intense quarantine and social distancing measures (4). COVID-19 has a higher hospitalization and mortality rate than influenza (5–7) and is spreading in an immune naive population (8). As of 30 November 2020, 61.8 million cases have been infected around the world counting over 1.4 million deaths (9). Moreover, there is increasing evidence that people with mild or no symptoms at the pre-symptomatic and early stages of infection can contribute to the spread of COVID-19 (10).

Since there is no effective treatment nor any vaccine for COVID-19, strategies for reducing the burden of the pandemic are focused on non-pharmaceutical interventions for reducing the spread of the infection, such as social-distancing measures, contact-tracing, quarantine, isolation, and the use of face masks in public (11). Public health policies promoting the use face masks in the community, i.e., in public places, can therefore have an important role in controlling the spread of the SARS-CoV-2 virus and for COVID-19 lockdown exit strategies (12). The published literature on the efficacy, effectiveness and acceptability of different types of face mask in preventing respiratory infections during epidemics is scarce and conflicting. However, face mask use is increasingly recommended and the potential of this intervention is not well-understood (13). National and international health organizations have adopted divergent policies on the subject. Recently, the CDC (Centers for Disease Control and Prevention) and the ECDC (European Center for Disease Prevention and Control) have advocated the use in public places of non-medical face mask (e.g., cloth mask) as a measure for the prevention and/or containment of SARS-CoV-2 infection (10, 14). In areas of significant community-based transmission, where it is difficult to maintain 6-feet social distancing (e.g., grocery stores and pharmacies), CDC recommends wearing cloth face coverings. CDC is additionally advising the use of simple cloth face coverings to slow the spread of the virus and help reduce the transmission of the virus from people who may be infectious without knowing it (14). The World Health Organization (WHO) conditionally recommends face mask use in the community for asymptomatic individuals in severe epidemics or pandemics in order to reduce transmission in the community (15) but it does not recognize its effectiveness in preventing infection (1).

Medical and non-medical face masks are used extensively by the general population in Asian countries, such as China, Singapore, South Korea, and Japan. Face mask wearing practice has been adopted since the 2003 SARS epidemic in addition to many other response measures and practices, including respiratory etiquette and hand hygiene (10). In Europe, as of 1 April 2020, Lithuania, Austria, Czechia, Slovakia, and Bulgaria recommend the use of face masks for persons going out in public (10).

Previous systematic reviews on the effectiveness of face mask use mainly focused on healthcare and household setting including only randomized controlled trials (RCTs) with most of them of low quality (16–19). We therefore conducted a systematic review of the existing scientific literature, with randomized trials and observational studies, including modeling and experimental studies, on the effectiveness and efficacy of wearing face masks in the community for reducing the spread of COVID-19 in non-healthcare and non-household setting.

## Aim

The aims of this systematic review (SR) were:

- to assess the efficacy and effectiveness of using masks in a community setting to reduce the spread of COVID-19 or other similar pandemic (20, 21); and in particular, to evaluate the effects of using vs. not using masks on mortality, infection rate and basic reproduction number (*R*_0_).
- to investigate the effect of different filtering capacity of masks used in community settings on the diffusion of the SARS- CoV2.

## Methods

The systematic review protocol was registered with the International Prospective Register of Systematic Reviews database (PROSPERO identifier: CRD42020184963). The study protocol and preliminary results are publicly available on https://osf.io/uvjgq. We conducted the systematic review following the preferred reporting items for systematic reviews and meta-analyses, the PRISMA statement (22), and the MOOSE guidelines for conducting meta-analysis of observational studies (23).

### Search Strategy

We searched for studies on the electronic databases MEDLINE, EMBASE, SCISEARCH, and The Cochrane Library from inception to April 22, 2020 using index terms related to face mask use in reducing spread of pandemic infection viruses. Gray literature was interrogated in MedRxiv, Rxiv, and bioRxiv databases. We hand searched the reference lists of the included papers. We also incorporated the studies included in any identified relevant systematic reviews. The full search strategy is reported in Supplementary Appendix 1.

### Eligibility Criteria

According to our PICOS questions (24), the following eligibility criteria without limit of study design were searched:

- Population: general population exposed to SARS-COV-2 infection or other similar virus (20, 21);
- Intervention and comparators: any type of mask such as non-medical face mask (i.e., cloth, gauze, tissue), medical face mask (i.e., surgical) and N95 respirators vs. no mask;
- Outcomes: mortality, respiratory infection rate (number of events) and the *R*_0_ of viral respiratory infections; filtering capacity of masks and viral load reduction.
- Setting: we defined “community-based setting” people of a group or unit that collectively sharing interests in the society for real life situations (e.g., schools, work, open spaces). Studies assessing the intervention in particular closed cluster setting exposed to higher risk of infection such as healthcare workers or households were excluded.

### Study Selection

Two reviewers independently screened the articles based on the titles, abstracts and full texts. The same two review authors independently retrieved and assessed full reports for potentially relevant studies for inclusion and exclusion according to the above criteria using a predefined electronic spreadsheet. In case of disagreement, consensus was achieved by involving a third independent review author. The reviewers' decisions and reasons for exclusion were recorded using appropriate reference management software such as EndNote. The study selection process was reported using the flow diagram of the Preferred-Reporting Items for Systematic Reviews and Meta-Analyses (PRISMA) (22).

### Data Extraction

Two reviewers independently extracted the study characteristic (e.g., first author, publication year, country, type of virus detected, study design, sample size, settings); for prognostic models, they extracted key characteristics (e.g., factors/predictors, time span, accuracy, and performance) and outcomes to be predicted. Disagreements were solved by consensus. A detailed data extraction form was developed prior to the systematic review being performed. In addition, for prediction modeling studies, the Checklist for critical Appraisal and data extraction for systematic Reviews of prediction Modeling Studies (CHARMS) was utilized (25).

### Primary and Secondary Outcomes

The primary outcomes of this systematic review are the following:

- Mortality rate;
- Respiratory infection rate (measured as event frequency), defined as fever ≥37.8°C with at least 1 respiratory symptom (sore throat, cough, sneezing, runny nose, nasal congestion, headache), with or without laboratory confirmation.
- *R*_0_ of viral respiratory infections;

The secondary outcomes were filtering capacity of masks and viral load reduction.

### Data Analysis

We examined the efficacy and effectiveness of wearing a mask and the models studies available in the literature by study design, setting, and study outcome. The data are summarized in both tabular and narrative formats. As the outcomes were dichotomous, such as respiratory infection, they were analyzed as pooled Risk Ratios (RRs), for unadjusted estimates. Adjusted odds ratios from multivariable regression reported in the studies were pooled as adjusted Odds Ratios (aORs). These are summarized using random effects meta-analysis using the DerSimonian and Laird random effects model (26), with heterogeneity calculated from the Mantel-Haenszel model. Due to the comprehensive definition of community-based setting, when possible, studies were sub-grouped based on study design and identified by appropriate setting to investigate potentially different effects on primary outcomes. If enough studies were present, we performed a sensitivity analysis of the primary outcomes selecting routine conditions for community-based setting excluding conditions at greater risk of gathering. All summary measures were reported with an accompanying 95% confidence interval. Data analyses were performed using RevMan Software.

### Assessment of Study Quality

Two independent reviewers appraised the risk of bias. In case of disagreement, a third reviewer was consulted. We used the Cochrane risk of bias tool for randomized controlled trials (27); the Newcastle Ottawa scale for non-randomized studies (28). We planned to use the PROBAST (Prediction model Risk Of Bias Assessment Tool) for Prediction Model Studies (29). However, since we found only quantitative-deterministic models, (statistical) bias was not a suitable measure of model goodness and we analyzed the QUAntitative-Deterministic models Risk of Infeasibility Assessment Checklist (QUADRIAC) according to the appropriate guideline (30). We provided more details in Supplementary Appendix 2.

### GRADE—Quality of the Evidence

The Grades of Recommendation, Assessment, Development and Evaluation (GRADE) framework for judging the quality of evidence has been extended to prognosis factor research. Evidence on prognostic models were evaluated by six factors that may decrease quality: (1) phase of investigation; (2) study limitations; (3) inconsistency; (4) indirectness; (5) imprecision; and (6) publication bias; and by two factors that may increase quality: (1) moderate or large effect size; and (2) exposure response gradient (31). Two independent reviewers graded the certainty of the evidence using the GRADE approach. Evidence was presented using GRADE Evidence Profiles developed in the GRADEpro (www.gradepro.org) software.

## Results

### Study Selection

**Figure 1:**
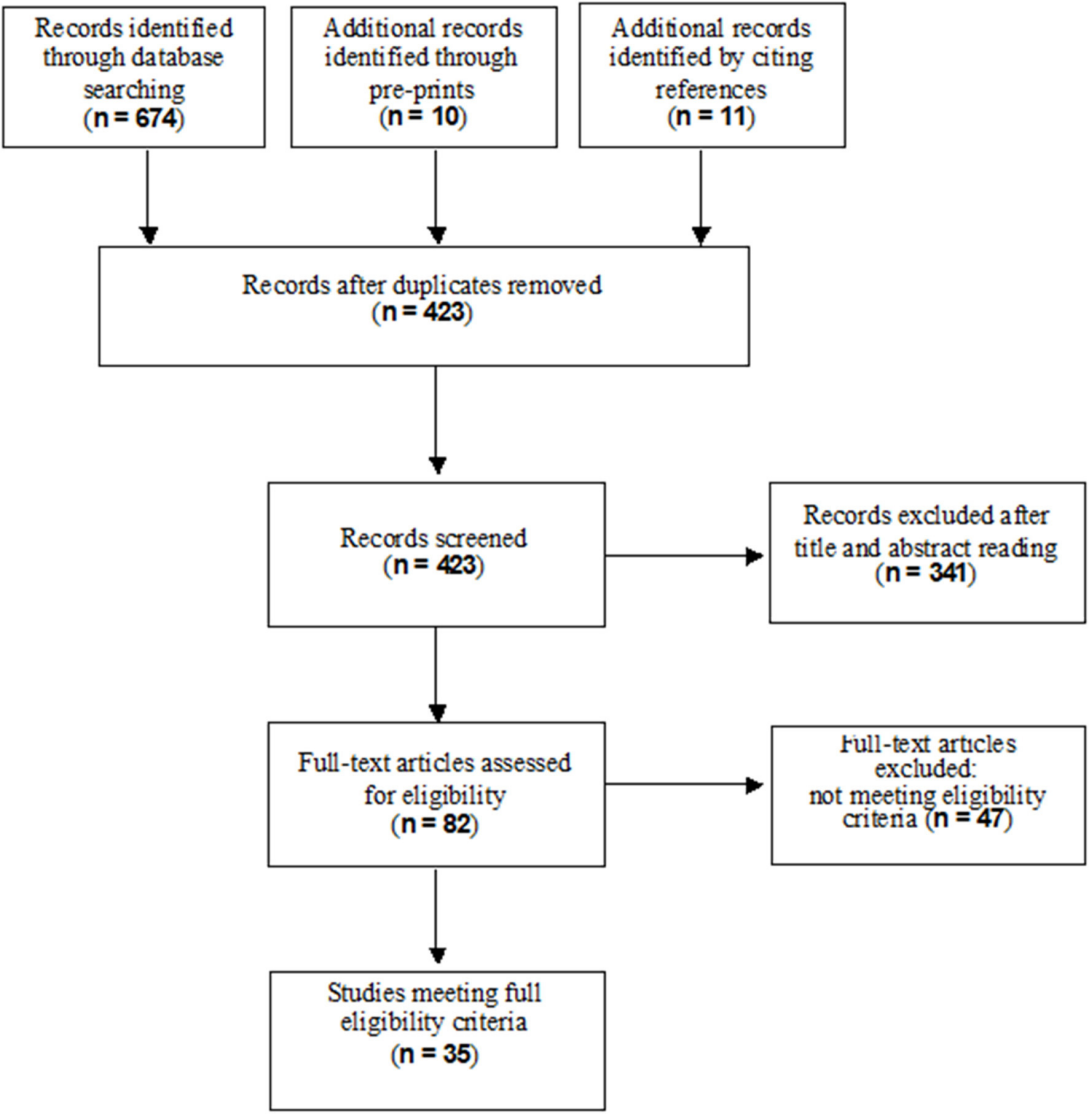
Flow diagram of study selection process.

A total of 684 records resulted from the searches in the electronic databases (MEDLINE, EMBASE, SCISEARCH) and from pre-prints; eleven additional records were identified through citations. After removing duplicates and excluding irrelevant records according to title, abstract and full text reading, 35 studies met our inclusion criteria for the final inclusion. Figure 1 shows the flow diagram of the study selection process.

### Description of the Included Studies

Table 1 reports characteristics of the included studies. Of the 35 included studies, three were cluster-RCTs (32, 33, 35), two cohort studies (38, 41), four were case-control (47, 51, 62, 64), four cross-sectional (34, 46, 49, 60), 13 were quantitative-deterministic predictive models (11, 13, 36, 39, 40, 42, 43, 45, 55, 57–59, 63), and nine were laboratory experimental studies (37, 44, 48, 50, 52–54, 56, 61).

**Table 1:** General characteristics of the included studies.

**References**	**Study design**	**Country**	**Setting**	**Population size (*n*)**	**Disease caused by virus**	**Adjusted Estimates**
Aiello et al. (32)	Cluster RCT	USA	University	930	H1N1 Influenza	Yes
Aiello et al. (33)	Cluster RCT	USA	University	816	H1N1 Influenza	Yes
Al-Jasser et al. (34)	Cohort	Saudi Arabia	Hajj pilgrimage	1,507	URTI	No
Alfelali (35)	Cluster RCT	Saudi Arabia	Hajj pilgrimage	7,687	vRTIs and CRI	Unclear
Babak (36)	SIR-based	Israel	Community	8 milions	COVID-19	-
Bae et al. (37)	Controlled Comparison	South Korea	COVID-19 patients	4	COVID-19	-
Balaban (38)	Cohort (pre-post survey)	Saudi Arabia	Hajj pilgrimage	186	H1N1 Influenza	No
Brienen et al. (39)	SIR-based	China	Community	Not reported	Influenza	-
Chen and Liao (40)	SIR-based	Taiwan	Nursery and primary school	494	Influenza	-
Choudhry (41)	Cohort	Saudi Arabia	Hajj pilgrimage	1,066	ARIs	No
Cui et al. (42)	SIR-based	not reported	Community	1 million	Influenza H1N1	-
D'Orazio et al. (43)	Agent-based	Italy	University campus	5,000	COVID-19	-
Davies et al. (44)	Controlled Comparison	UK	Healthy volunteers	21	Influenza (represented by Bacteriophage MS2 and B atrophaeus)	-
De Kai et al. (45)	SIR-based + Agent-based	38 selected countries (Asia, Europe and North America)	Community	not reported	COVID-19	-
Deris (46)	Cross-sectional	Malaysia	Hajj pilgrimage	387	ARIs	No
Eikenberry et al. (13)	SIR-based	USA	Community	1 million	COVID-19	-
Emamian, (47)	Nested case-control	Iran	Hajj pilgrimage	338	RTI (all types)	Unclear
Guha et al. (48)	Bench tests	USA	Laboratory	n.a.	Viral infection (Submicron-sized aerosols)	-
Kim (49)	Cross-sectional	South Korea	Primary school	7,448	H1N1 Influenza	Unclear
Lai et al. (50)	Controlled Comparison (2 manikins)	China	Laboratory	n.a.	Airborne infections	
Lau et al. (51)	Case-control	China	Community	990	SARS	Yes
Li et al. (52)	*In vivo* experiments	Hong Kong	Laboratory	10	Viral respiratory infection	-
Makison Booth et al. (53)	Bench test (Head of dummy test)	UK	Laboratory	n.a.	H1N1 and H5N1 influenza (vaccine strain of A-type virus simulation)	-
Milton et al. (54)	*In vivo* experiment	USA	Laboratory	37	Seasonal influenza	-
Mniszewski et al. (55)	Agent-based	USA	Community	20 millions	Influenza H1N1	-
Ngonghala (11)	SIR-based	USA	Community	19.45 millions	COVID-19	-
Rengasamy et al. (56)	Bench test	USA	Laboratory	n.a.	Influenza	-
Tian et al. (57)	SIR-based	China	Community	159	COVID-19	-
Tracht et al. (58)	SIR-based	USA	Community	1 million	Influenza H1N1	-
Tracht et al. (59)	SIR-based	USA	Community	302 millions	Influenza H1N1	-
Uchida (60)	Cross-sectional	Japan	Community	10,524	Influenza	Unclear
van der Sande et al. (61)	Bench test	Netherlands	Laboratory	61	Influenza	-
Wu (62)	Case-control	China	Community	375	SARS	Unclear
Yan et al. (63)	SIR-based	USA	Community	Not reported	Influenza	-
Zhang et al. (64)	Case-control	USA-China	Airplane	9	H1N1 Influenza	No

Of the 13 epidemiological studies (RCTs and observational studies) included in the review, four were carried out in a university (32, 33) or school setting (49, 60), one on an airplane (64), six during mass gatherings (34, 35, 38, 41, 46, 47), and two in non-specific community settings (51, 62).

As far as the quantitative-deterministic models are concerned, ten studies developed a SIR-based model (11, 13, 36, 39, 40, 42, 57–59, 63), two studies developed an agent-based model (43, 55), and one study employed both (45). The whole population was considered in all the studies but one, which was restricted to nursery and primary school children (40). Three out of the 13 modeling studies (40, 43, 45) considered a closed environment; three models (45, 57, 63) contemplated both inward and outward filtering capacity whereas the others did not make such a distinction; furthermore, only two among the reviewed studies have accounted for a proper usage of facial masks (13, 57). It is worth mentioning that five out of the 13 studies that have analyzed a quantitative-deterministic model have also accounted for the intervention timing (11, 13, 42, 45, 59). Four studies across all the reviewed studies were pre-prints (11, 24, 43, 57).

The laboratory experimental studies were highly heterogeneous in terms of setting/participants: four bench test (48, 53, 56, 61), two *in vivo* studies (52, 54), and three controlled studies (37, 44, 50).

Supplementary Appendix 2 lists included and excluded studies.

### Risk of Bias of Epidemiologic Studies and Unfeasibility of Deterministic Models

Focusing on randomized trials, we found high risk of performance and detection bias. However, blinding of participants was not possible due to the nature of the interventions. The included trials were characterized by an overall high quality. Among observational studies the quality ranged from poor to fair for cohort and case-controls studies, whereas it ranged from fair to good for cross-sectional studies. Focusing on mathematical models, we evaluated the unfeasibility of quantitative-deterministic models reporting eight of 13 studies with medium overall risk of infeasibility (two high and three low). Supplementary Appendix 3 lists the risk of bias of epidemiologic studies and unfeasibility of deterministic models.

### Outcomes

Although no epidemiologic study on wearing face masks in the community for reducing the spread of COVID-19 has been published, a number of studies gave an indirect estimate of the protective efficacy of masks for other viral respiratory infections from agents similar to SARS-CoV2.

#### Mortality Rate

##### Deterministic Models

Four out of 13 quantitative-deterministic models reported data on mortality (13, 36, 45, 59). Among them, only one study (59) has explicitly provided quantitative data in three scenarios based on different initial values of *R*_0_; however, the time horizon was not specified. Three studies (13, 36, 45) presented graphs depicting the evolution over time of cumulative deaths. Overall the studies point toward a reduction in mortality when the population mask coverage is near-universal, regardless of mask efficacy.

Table 2 describes the mortality in relation to the initial R0, type of mask, mask filtration efficacy (%) and adherence of population coverage (%). Summary of findings (SOF) are displayed in Table 3.

**Table 2:** Mortality rate in the quantitative-deterministic models.

	**Intial R_0**	**Type**	**Efficacy %**	**Population coverage %**	**Deaths**
Tracht et al. (59)	1.25	N95	20	0	286,236
				10	26.445
				25	2.468
				50	1.730
	1.30	N95	20	0	327.270
				10	56.280
				25	8.301
				50	2.027
	1.35	N95	20	0	356.462
				10	84.131
				25	8.301
				50	2.573
Eikenberry et al. (13)	Extraction not possible—only graphs				
De Kai et al. (45)	Extraction not possible			0	Not reported
				50	240.000
				80	60.000
Babak (36)	2.2	Not reported	8	Near-universal	Extraction not possibile
			16	(% not reported)	
	1.3	Not reported	8	Near-universal	Extraction not possibile
			16	(% not reported)	

**Table 3:** Summary of findings.

**Wearing a mask compared to no mask in a community setting**						
**Patient or population**: community						
**Intervention**: mask wearing						
**Comparison**: no mask wearing						
**Outcomes**		N**°of participants** ** (studies)** ** Follow up**	**Certainty of the evidence** ** (GRADE)**	**Relative effect** ** (95% CI)**	Anticipated absolute effects^*^	
					**Risk with no mask wearing**	**Risk difference with mask wearing**
**Mortality rate**					The general consensus points toward a reduction of deaths when the population mask coverage is near-universal, regardless of mask efficacy.	
**Respiratory infection**	In randomized controlled trials	4017 (3 RCTs)	⊕◯○○ VERY LOW ^a^^,^^b^^,^^c^	**RR 0.97** (0.72 to 1.31)	112 per 1,000	**3 fewer cases per 1,000** (31 fewer to 35 more)
	In cross sectional studies	16,413 (four observational studies)	⊕○○○ VERY LOW ^b^^,^^d^	**RR 0.90** (0.74 to 1.10)	172 per 1.000	**17 fewer per 1.000** (45 fewer to 17 more)
	In case-control studies	1,501 (fourobservational studies)	⊕○○○ VERY LOW ^b^^,^^d^^,^^e^	**RR 0.59** (0.34 to 1.03)	405 per 1.000	**166 fewer per 1.000** (267 fewer to 12 more)
	In prospective studies	960 (two observational studies)	⊕○○○ VERY LOW ^b^^,^^f^^,^^g^^,^^h^	**RR 0.55** (0.11 to 2.75)	584 per 1.000	**263 fewer per 1.000** (520 fewer to 1,022 more)
Basic reproduction number (R0) of viral respiratory infection					In the worst-case scenario with a mask efficacy at 30% and a population coverage at 20%, the *R*_0_ reduced from the initial value of 2.0 to just 1.9 whereas in the best-case scenario when the mask efficacy is at 95%, the *R*_0_ can fall to 0.99 from an initial value of 16.90, even though no population neither coverage nor time horizon are reported	
Filtering capacity of masks		Seven experimental laboratory studies	⊕○○○ VERY LOW^i^		High degree of variation of filtration efficiency depending on the materials used. All types of masks might reduce aerosol exposure. However, personal respirators were more efficient than surgical masks, which were more efficient than home-made masks.	
Viral load reduction		One experimental laboratory study (37 volunteers)	⊕○○○ VERY LOW i	**RR 0.25 (0.09 to 0.67)**	432 per 1.000	**324 fewer per 1.000** (394 fewer to 143 fewer)
*The risk in the intervention group (and its 95% confidence interval) is based on the assumed risk in the comparison group and the **relative effect** of the intervention (and its 95% CI). **CI:** Confidence interval; **OR:** Odds ratio						
**GRADE Working Group grades of evidence** **High certainty:** We are very confident that the true effect lies close to that of the estimate of the effect **Moderate certainty:** We are moderately confident in the effect estimate: The true effect is likely to be close to the estimate of the effect, but there is a possibility that it is substantially different **Low certainty:** Our confidence in the effect estimate is limited: The true effect may be substantially different from the estimate of the effect **Very low certainty:** We have very little confidence in the effect estimate: The true effect is likely to be substantially different from the estimate of effect						

a *High risk from multiple bias*.

b *Not COVID-19 population*.

c *The line of “no difference” included important benefit and harms*.

d *I^2^>75%*.

e *Ascertainment of exposure*.

f *Wide confidence intervals comprising important benefit and harm*.

g *Ascertainment of exposure and assessment of the outcome*.

h *I^2^>90%*.

i *Experimental study, high variability in type of masks equipment*.

#### Respiratory Infection Rate

##### RCT

**Figure 2:**
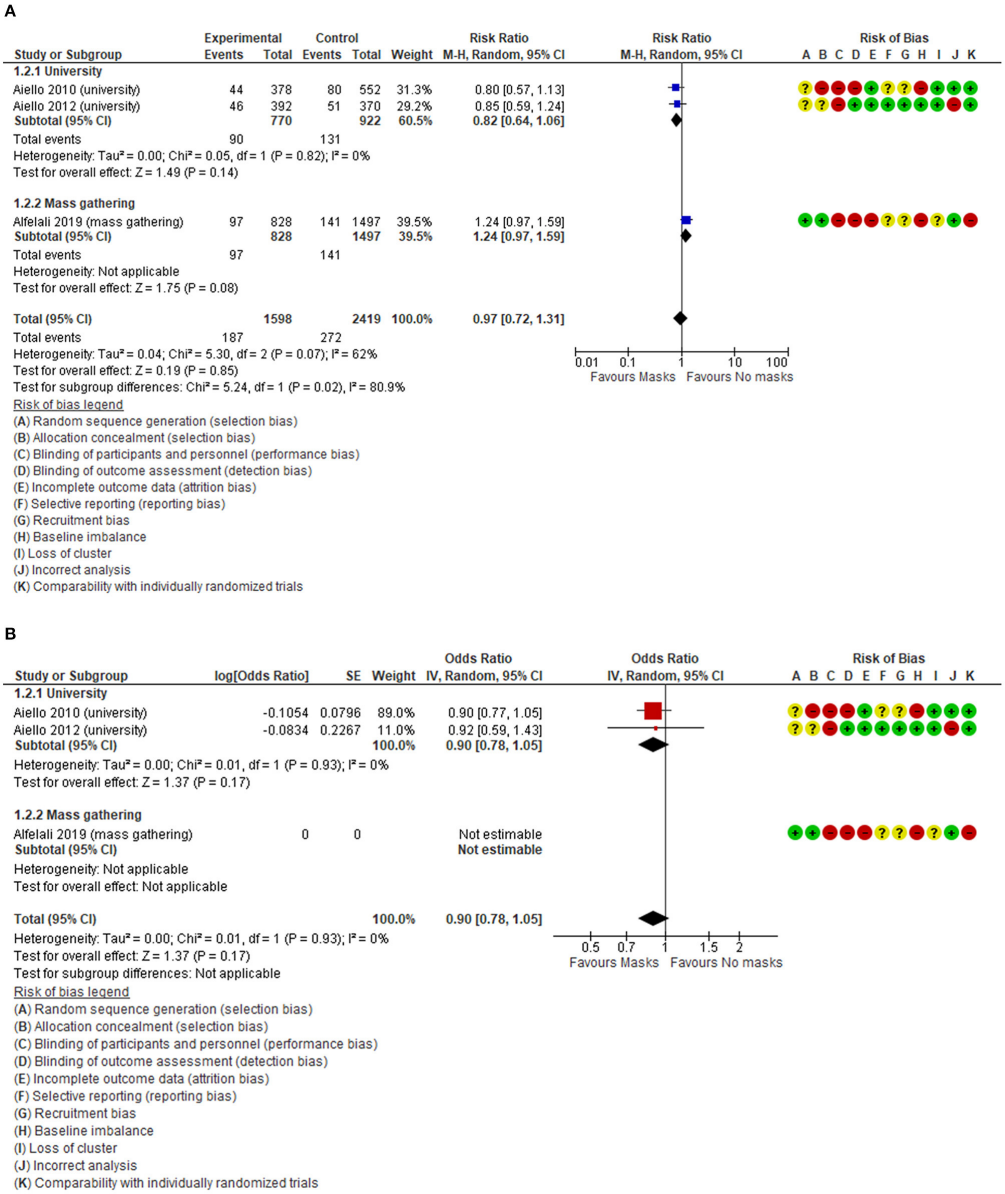
**(A)** Unadjusted forest plot of respiratory infection rate and risk ratios in RCTs. **(B)** aORs forest plot of respiratory infection rate in RCTs.

The overall findings were similar between adjusted and unadjusted estimates. With very low quality of the evidence (Table 3), in the unadjusted data, three cluster-RCTs (32, 33, 35) have reported a small non-significant reduction in the risk of respiratory infections (Figure 2A, RR 0.97, 95% CI 0.72–1.31, *I*^2^ = 62%). The adjusted estimates of two, out of three, cluster-RCTs (32, 33) confirmed the reduction with high consistency, even if not at statistically significant levels (Figure 2B, aOR 0.90, 95% CI 0.78–1.05, *I*^2^ = 0%).

##### Observational Studies

**Figure 3:**
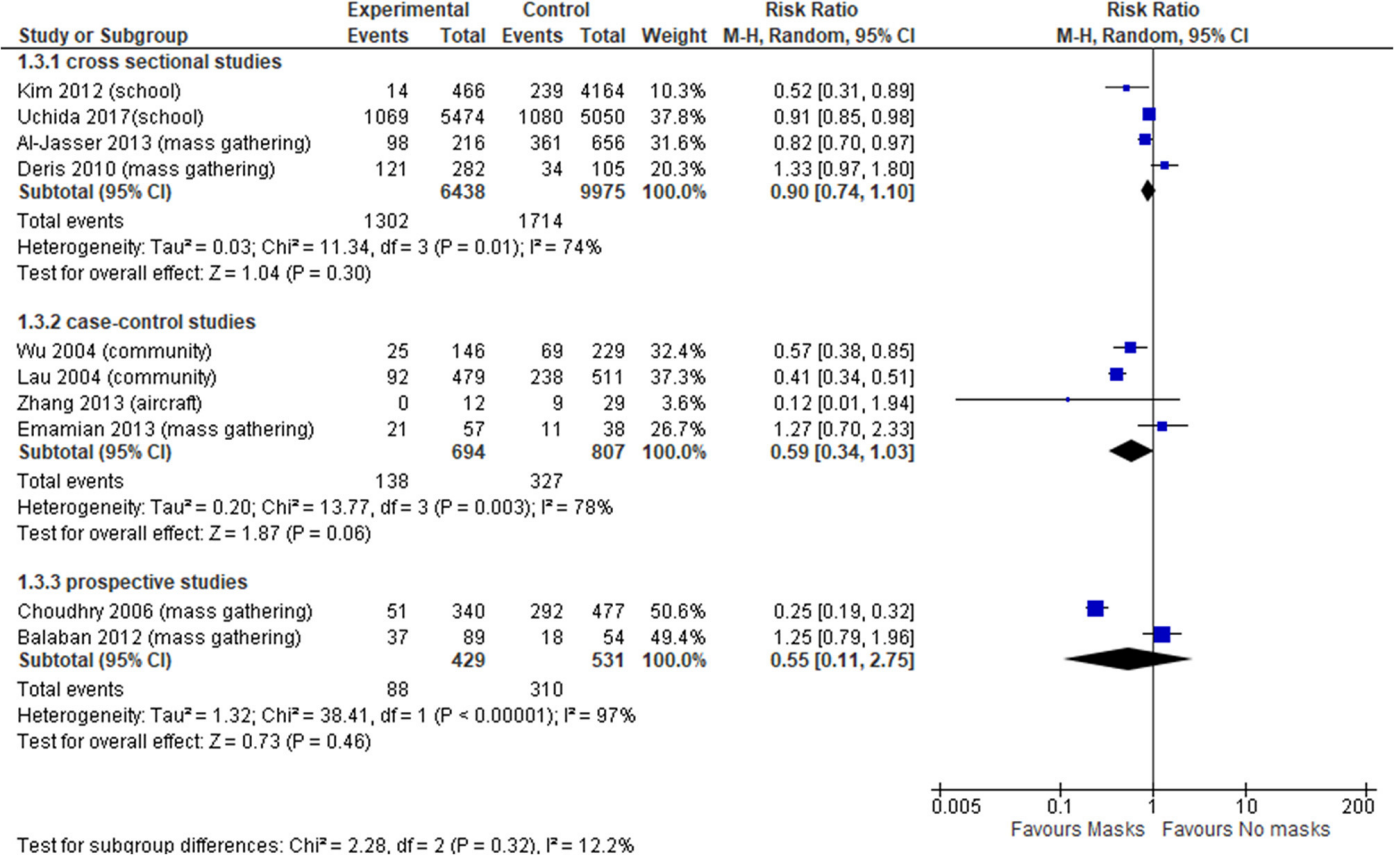
Forest plot of respiratory infection rate in observational studies.

In total, 10 observational studies were identified (34, 38, 41, 46, 47, 49, 51, 60, 62, 64), among which one study reported adjusted data in relation to the outcome of interest (51). Thus, the meta-analysis was reported only for unadjusted estimates. The level of certainty of the evidence in all observational studies was very low (Table 3), with no statistically significant effect; the overall effect was very imprecise across all cross sectional studies (four studies, OR 0.90, 95% CI 0.74–1.10, *I*^2^ = 74%) (Figure 3, Comparison 1.3.1) (34, 46, 49, 60), case-control (four studies, OR 0.59, 95% CI 0.34–1.03, *I*^2^ = 78%) (Figure 3, Comparison 1.3.2) (47, 51, 62, 64) and prospective cohort studies (two studies, OR 0.55, 95% CI 0.11–2.75, *I*^2^ = 97%) (Figure 3, Comparison 1.3.3) (38, 41).

Since we found high heterogeneity, we performed sensitivity analysis excluding aircraft and mass gathering studies to determine the robustness of our original analyses and determine whether special settings might have influenced the overall pooled effect. Focusing on studies set in schools, universities and in the general community, the evidence from cross-sectional studies was not statistically significant (OR 0.74, 95% CI 0.43–1.26, *I*^2^ = 76%) (Supplementary Appendix 4, Supplementary Figure 2); whereas case-control studies (OR 0.46, 95% CI 0.34–0.62, *I*^2^ = 47%) (Supplementary Appendix 4, Supplementary Figure 2) showed a statistically significant effect in favor of wearing face masks vs. not wearing masks, with a more precise overall estimate.

##### Deterministic Models

Ten out of 13 studies that deployed mathematical models examined respiratory infection rates (11, 13, 36, 39, 40, 42, 43, 55, 57–59, 63). Across them, three studies (39, 43, 55) reported such an outcome as a pure rate; two papers (42, 58) reported results about the respiratory infections rate as the percentage of cumulative cases; one study (59) reported the number of cumulative cases. The remaining studies did not report intelligible results on respiratory infection rate. Across the above-mentioned studies, the ones that reported the use of N95 masks agree that when at least 50% of the population is wearing a mask the respiratory infection rate can be reduced by a percentage ranging from 80% up to 99%.

The use of facial mask results in a reduction of the respiratory infection rate that is at least of 2.0% in the worst case scenario (58) and up to 99% in the best case scenarios (39, 58, 59). No time-horizon is specified, though Supplementary Appendix 5, Supplementary Table 9. The summary of Findings is displayed in Table 3.

#### Basic Reproduction Number (**R**_**0**_) of Viral Respiratory Infections

##### Deterministic Models

Seven out of 13 studies that deployed a mathematical model investigated the *R*_0_ of viral respiratory infections (11, 39, 40, 42, 55, 57, 58). Across them, four papers reported explicitly the results (40, 42, 51, 57), two papers only reported a graph (11, 39), and the remaining one did not report accessible data (55). Across these studies, the worst-case scenario was reported in the Brienen et al. (39): with mask efficacy at 30% and a 20% population coverage, the *R*_0_ reduced from the initial value of 2.0 to just 1.9. On the other hand, the best-case scenario is reported in Chen (40): with mask efficacy at 95%, the *R*_0_ can fall to 0.99 from an initial value of 16.90; however, neither population coverage nor time horizon are reported. Supplementary Appendix 5, Supplementary Table 9 show the description of *R*_0_ of viral respiratory infections across models. The summary of Findings is displayed in Table 3.

#### Filtering Capacity of Masks

Among laboratory experimental studies, seven out of nine studies reported the outcome as filtration rate or face mask protection, including goodness-of-fit and filtration efficiency. Outcomes varied according to the materials used.

In adults, generally filtration rate of household materials had high degree of variation, ranging from 49 to 86% for 0.02 μm exhaled particles (44) and from 3 to 60% for particles in the relevant size range (56).

High degree of variation were also present in surgical masks. One study (56) reported a filtration rate of surgical masks comparable to that of masks made of household materials. Other studies reported the best peformance for surgical masks, filtering from 89% (44) to 95.5–97% (52) of small particles. Under a pseudo-steady concentration environment, face mask protection on average was found to be 45%, while under expiratory emissions, protection varied from 33 to 100% for fully sealed face mask (50).

Particularly, in pediatrics, penetration of neutralized polydispersed sodium chloride aerosols varied significantly between brands at the highest flow rates, from 15 to 50% (48).

All types of surgical masks provided a relatively stable reduction of aerosol exposure over time, unaffected by duration of wear or type of activity, but with a high degree of individual variation with reductions ranging from 1.1- to 55-fold (average 6-fold), depending on the design of the mask (53). One study compared all types of masks (N95 personal respirators, surgical and home-made masks): surgical masks provided about twice as much protection as home-made masks, with the difference being slightly more marked among adults. N95 personal respirators provided adults with about 50 times as much protection as home-made masks, and 25 times as much protection as surgical masks (61). The summary of Findings is displayed in Table 3.

#### Viral Load Reduction

Three experimental laboratory studies were included (37, 44, 54), of which one study having three arms investigating surgical masks, home-made masks (i.e., cotton mask) or no mask; two studies focussed on the comparisons between surgical masks vs. home-made masks or no mask (Supplementary Appendix 5, Supplementary Table 10). According to our PICO, for the SOF GRADE assessment only the comparison between surgical mask vs. no mask reporting outcome data was considered (54). This suggested a viral load reduction of 0.25 (0.09–0.67) in favor of face mask use (risk difference: 324 fewer × 1,000) (Table 3).

## Discussion

We found very low-certainty evidence that wearing a face mask is associated with a reduced risk of primary infection in RCTs as well as in observational studies. However, the wide confidence intervals affected the statistical significance of the overall estimate. It was not possible to establish the certainty of evidence about mortality, filtering capacity and *R*_0_ whereas viral load was judged to be of very low quality. Our findings indicate (i) a general consensus toward a reduction of deaths, based on prediction modeling studies, when the population mask coverage is near-universal, regardless of mask efficacy; (ii) filtration efficiency depends on the face mask materials, with studies showing high variability. It seems that all types of masks reduce the viral exposure, even though the levels of protection, in terms of reduction of susceptibility to infection in the wearer, are probably lower for some materials (i.e., cloth masks), to the extent that they do not effectively protect against infectious aerosols. Specifically, personal respirators were more efficient than surgical masks, which were more efficient than home-made masks; (iii) in the worst-case scenario with a mask efficacy at 30% and a population coverage at 20%, the *R*_0_ reduced from the initial value of 2.0 to just 1.9; whereas in the best-case scenario, when the mask efficacy is 95%, the *R*_0_ can fall to 0.99 from an initial value of 16.90, even though no population coverage nor time horizon is reported; (iv) wearing vs. not wearing a mask is associated with a reduction of viral load of RR 0.25 (95% CI 0.09–0.67, based on one experimental laboratory study).

Overall, our findings support the recommendation on using face masks in community settings in a pandemic era: home-made masks, such as those made of teacloths, may confer a significant degree of protection, albeit less strong than surgical masks or N95 personal respirators. Mask efficacy at 95% (N95 personal respirators) seems to be the best scenario, but it is difficult to realize in terms of adherence and costs from a public health perspective. A balanced compromise in the community could be reached with high population coverage using surgical masks (whose mask efficacy is >95%), which is easier to implement. Comparing surgical masks to no mask has shown a viral load reduction of a quarter (risk difference: 324 fewer × 1,000). Surgical masks were more effective than homemade masks in reducing the number of microorganisms expelled. However, high levels of filtration efficiency have been found among surgical and non-surgical masks, with evidence from all experimental laboratory studies emphasizing the importance of high filtration capacity irrespective of the materials used.

Our findings are in line with results from previous systematic reviews, which however had different aims, population and outcomes. For example, examining the infection rate in pandemic influenza transmission Jefferson et al. (65) has shown that wearing masks significantly decreased the spread of SARS (OR = 0.32; 95% CI 0.25–0.40). Similar finding were found in studies on respiratory virus infections including SARS, H1N1, and COVID-19 in all subgroups, including non-health care worker or non-household contacts (66). One review, investigating the optimum use of different personal protective equipment (face masks, respirators, and eye protection) in community and health-care settings, reported a large reduction in the risk of infection in favor of face mask use (OR 0.15; 95% CI 0.07 to 0.34, RD −14.3%; −15.9 to −10.7; low certainty), with stronger associations with N95 or similar respirators, compared with disposable surgical masks or similar masks (67). Anyway, this is the first systematic review and meta-analysis comprising evidence based on different research methods and study designs (e.g., modeling studies), to address the existing uncertainty about the efficacy and effectiveness of wearing a mask targeting the community setting for limiting the spread of COVID-19.

A pragmatic ecologic study, involving 49 countries, used data from the European Center for Disease Prevention and Control (ECDC) and investigated the association between face mask use in the community and cumulative number of cases of COVID-19 infection per million inhabitants, discovering that face mask use was negatively associated with number of COVID-19 cases (coef. −326; 95% CI −601 to −51, *P* = 0.021) (68).

The results of this ecological study and of the individual-level studies included in the review are in line with our findings, supporting the use of face masks for reducing the transmission and acquisition of respiratory viral infections in the community.

### Strength and Limitations

Our review included experimental laboratory research and mathematical modeling studies to complement observations studies and trials, for obtaining a more complete picture on mortality and viral load reduction, filtering capacity and population coverage which are important factors influencing the *R*_0_. We adopted full methodological rigor within a much shorter time-frame compared to traditional reviews, using enhanced processes. We also critically assessed the risk of bias of included studies (randomized controlled studies and observational studies) and infeasibility of mathematical modeling studies.

Our systematic review has some limitations. Only a minority of the included studies looked at COVID-19, mainly addressed by modeling studies. We did not investigate the balance of pros and cons of wearing a mask. On one hand, the use of face masks may provide a false sense of security leading to suboptimal physical distancing, poor respiratory etiquette and hand hygiene—and possibly not staying at home when ill. There is a risk that improper removal of the face mask, handling of a contaminated face mask or an increased tendency to touch the face while wearing a mask by healthy persons might actually increase the risk of transmission (10). On the other hand, the fears related to the paradoxical increase of the infectious risk for their improper use are entirely theoretical, based on preconception without real foundation. Education campaigns should be encouraged for assuring proper use (10).

We reported adjusted estimates from two out of three cluster RCTs, because one RCT (35) might have unreliable results due to low usage of face masks in participants: indeed, a low usage of masks was reported in the face mask group, with adherence of only 25% among participants. In contrast, a moderate proportion of participants in the control group (49%) used face masks daily and intermittently. This undermines the reliability of results. We performed sensitivity analysis in order to present routine situations in the community but the included three places (schools, universities and in the general community) have different characteristics (e.g., open/closed space, potential confounder/interaction variable).

We did not appraise the quality of laboratory experimental studies since we did not find appropriate tools for measuring it. Similarly, for mathematical models we used the unfeasibility appraisal, a proxy of quality assessment, which is more appropriate given the nature of the studies. As for the quantitative-deterministic studies, we acknowledge that such models, especially when SIR-based, do not provide estimates of events, but rather describe what could happen in the future with respect to a predefined set of initial conditions. Namely, they help stakeholders in understanding how the situation could evolve in the future if different actions are adopted today. It follows that pitfalls of such models can be due to mis-specified initial conditions.

With new publications on COVID-19 related prediction models rapidly entering the medical literature, this systematic review cannot be viewed as an up to date list of all currently available prediction models. Furthermore, there were some studies among the ones we have reviewed that were available only as preprints; such studies might actually bring new insights after the peer-reviewing process.

### Challenges and Opportunities for Public Health

The speed of the worldwide spread of the SAR-COV-2 virus, leading to a severe pandemic for which there is no effective treatment or vaccine and limited knowledge on disease behavior, and the uncertainty regarding the role of asymptomatic individuals in the transmission of the virus, call for Public Health infection prevention and control measures, even in the absence of evidence or in the presence of low quality scientific evidence. A recent systematic review found that, in this pandemic, the proportion of asymptomatic cases ranged from 4 to 41% (69). In this light, universal masking in the community may mitigate the extent of transmission of COVID-19 and may be a necessary adjunctive public health measure (70).

The evidence-based medicine should be used with acumen. The evidence-based GRADE approach suggest that whenever the evidence in favor to the intervention is low but the risks related to the averted implementation could be high, drastic measures can be adopted even in the absence of solid evidence, if the conditions are met (71).

The SARS-COV-2 pandemic is a life-threatening condition to such an extent as to indicate the need of accepting a minimal risk, assuming there is such a risk (i.e., mask costs), of the community intervention (i.e., face mask use), considering the notable benefit of its implementation, even if the evidence-base is of low quality. Deferring these measures, on the other hand, can have a negative effect on health policy decisions. This is called “precautionary principle”: “when human activities may lead to morally unacceptable harm that is scientifically plausible but uncertain, actions shall be taken to avoid or diminish that harm” (72, 73). The evidence, albeit imperfect, in support of the use of masks in this context are justifiable and sufficient in light of this principle. Although evidence-based medicine rightly looks suspiciously at tests of low methodological quality, at the same time it does not completely dismiss them, when circumstances are appropriate, as in this case (8).

And, when it comes to parachuting from a plane that is crashing, you wear it even if no trial has ever shown its effectiveness compared to a control group that launched without (74).

It should be emphasized that the use of face masks in the community should be considered only as a complementary measure and not as a replacement for the core preventive measures that are recommended to reduce community transmission including physical distancing, staying home when ill, teleworking/home working if possible, respiratory etiquette, meticulous hand hygiene and avoiding touching the face, nose, eyes, and mouth (10). In conclusion, the use of face masks as single intervention is not sufficient to stop the spread of COVID19 and a full package of the above mentioned interventions is the safest and the most recommended approach.

## Data Availability Statement

Data can be found in Supplementary Material and the dataset is stored at the following repository https://osf.io/uvjgq/.

## Author Contributions

DC and AN carried out the literature search, conducted screenings, extracted data, and completed the risk of bias assessment. AF, LI, DDA, and CR provided a critical revision of the manuscript. SG and GC conceived and drafted the manuscript and performed the statistical analyses. ADM extracted data and assessed quality of modeling studies. PI conceived and drafted the manuscript, interpreted the data, wrote the discussion, and guarantor of the data. CM and GT interpreted the data and revised the manuscript for important intellectual content. All authors approved the final version of the manuscript.

## Conflict of Interest

The authors declare that the research was conducted in the absence of any commercial or financial relationships that could be construed as a potential conflict of interest.

## Abbreviations

aOR: Adjusted Odds Ratio
CI: Confidence Interval
COVID-19: Coronavirus disease 2019
CDC: Centers for Disease Control and Prevention
CHARMS: Checklist for critical Appraisal and data extraction for systematic Reviews of prediction Modeling Studies
ECDC: European Center for Disease Prevention and Control
GRADE, Grades of Recommendation, Assessment: Development and Evaluation
MERS: Middle East respiratory syndrome
MOOSE: Guidelines for Meta-analysis and Systematic reviews of observational studies
NOS: Newcastle Ottawa scale for non-randomized studies
PRISMA: Preferred reporting items for systematic reviews and meta-analyses
PROBAST: Prediction model Risk Of Bias Assessment Tool
RCT: Randomized Controleld Trials
RR: Risk Ratio
QUADRIAC: QUAntitative-Deterministic models Risk of Infeasibility Assessment Checklist
SARS: severe acute respiratory syndrome
SARS-CoV-2: severe acute respiratory syndrome coronavirus-2
SR: Systematic Reviews
SOF: Summary of Findings
WHO: World Health Organization.
