# Supplementary material for: Face Mask Use in the Community for Reducing the Spread of COVID-19: A Systematic Review
Source: Front Med (Lausanne). 2021 Jan 12;7:594269. doi: 10.3389/fmed.2020.594269 (PMC7835129; doi:10.3389/fmed.2020.594269)
Supplement: Supplementary file 1 [file Data_Sheet_1.docx]

**Facemasks in Community for Reducing the Spread of COVID-19: a systematic review**

*Daniela Coclite, Antonello Napoletano, Silvia Gianola_,_ Andrea del Monaco, Daniela D’Angelo, Alice Fauci, Laura Iacorossi, Roberto Latina, Giuseppe La Torre, Claudio M. Mastroianni, Cristina Renzi, Greta Castellini, Primiano Iannone*

**Supplementary** **contents**

[**Appendix 1. Search strategies** 2](#_Toc48170230)

[Table S1- Search strategy 2](#_Toc48170231)

[**Appendix 2. Included studies and excluded studies** 3](#_Toc48170232)

[Table S2- Included studies 3](#_Toc48170233)

[Table S3 - Excluded studies 5](#_Toc48170234)

[**Appendix 3. Risk of bias of epidemiologic studies and unfeasibility of deterministic models** 8](#_Toc48170235)

[Figure S1- Risk of bias in randomized controlled trials 8](#_Toc48170236)

[Table S4- Methodological quality of cohort studies 9](#_Toc48170237)

[Table S5- Methodological quality of case-control studies 9](#_Toc48170238)

[Table S6 - Methodological quality of cross-sectional studies 10](#_Toc48170239)

[Table S7 – QUAntitative-Deterministic models Risk of Infeasibility Assessment Checklist (QUADRIAC) 11](#_Toc48170240)

[Table S8 - Unfeasibility of deterministic models 13](#_Toc48170241)

[**Appendix 4. Sensitivity analyses** 14](#_Toc48170242)

[Figure S2 - Sensitivity excluding aircraft and mass gathering studies. 14](#_Toc48170243)

[**Appendix 5. Additional analysis** 15](#_Toc48170244)

[Table S9. Outcomes Models 15](#_Toc48170245)

[Table S10.Viral load reduction 16](#_Toc48170246)

[**Appendix 6. PROSPERO protocol registration** 17](#_Toc48170247)

[**Appendix 7. Reporting checklists** 21](#_Toc48170248)

[Table S11 - PRISMA checklist 21](#_Toc48170249)

[Table S12- MOOSE checklists 24](#_Toc48170250)

[**Appendix 8. References of Supplements** 26](#_Toc48170251)

## **Appendix 1. Search strategies**

# Table S1- Search strategy

1. FILE **'MEDLINE, EMBASE, SCISEARCH**' ENTERED AT 15:45:14 ON 22 APR 2020
2. L4 8571 S COVID 19/CT,TI,AB OR COVID19/CT,TI,AB
3. L5 79 S SARS COV2/CT,TI,AB
4. L6 2341 S SARS COV 2/CT,TI,AB
5. L7 1254 S NCOV/BI OR N COV/CT,TI,AB
6. L8 3523 S NOVEL CORONAVIRUS/CT,TI,AB OR NOVEL CORONA VIRUS/CT,TI,AB
7. L9 556 S NEW CORONAVIRUS/CT,TI,AB OR NEW CORONA VIRUS/CT,TI,AB
8. L10 2517 S CORONAVIRUS DISEASE#/CT,TI,AB OR CORONA VIRUS DISEASE#/CT,TI,
9. L11 2302 S SEVERE ACUTE RESPIRATORY SYNDROME CORONAVIRUS 2/CT,TI,AB
10. L12 10 S SEVERE ACUTE RESPIRATORY SYNDROME CORONA VIRUS 2/CT,TI,AB
11. L13 79500 S PANDEMIC/CT,TI,AB
12. L14 18756 S PANDEMIC INFLUENZA/CT,TI,AB
13. L15 790 S PANDEMIA#/CT,TI,AB
14. L16 49859 S INFLUENZA, HUMAN/CT,TI,AB
15. L17 104202 S INFLUENZA/BI AND HUMAN+NT/CT
16. L18 214876 S L4-L17
17. L19 4071 S FACEMASK#/CT,TI,AB
18. L20 9752 S FACE# MASK#/TI,AB
19. L21 1626 S SURGICAL MASK#/CT,TI,AB
20. L22 1 S COMMUNITY MASK#/TI,AB
21. L23 55 S CLOTH MASK#/CT,TI,AB
22. L24 571 S PROTECT? MASK#
23. L25 26 S FFP1/TI,AB
24. L26 86 S FFP2/TI,AB
25. L27 68 S FFP3/TI,AB
26. L28 752 S L18 AND L19-L27
27. L29 736 S L28 AND (ENGLISH/LA OR FRENCH/LA OR SPANISH/LA OR ITALIAN/LA
28. L30 674 S L29 NOT (EDITORIAL/DT OR NEW/DT OR LETTER/DT OR COMMENT?/DT)
29. L31 402 DUP REM L30 (272 DUPLICATES REMOVED)

## **Appendix 2. Included studies and excluded studies**

# Table S2- Included studies

| Aiello AE et al. Facemasks, hand hygiene, and influenza among young adults: a randomized intervention trial. PLoS One. 2012;7(1):e29744. doi: 10.1371/journal.pone.0029744. |
| --- |
| Aiello AE et al. Mask Use, Hand Hygiene, and Seasonal Influenza-Like Illness among Young Adults: A Randomized Intervention Trial. J Infect Dis. 2010 Feb 15;201(4):491-8. doi: 10.1086/650396. |
| Al-Jasser FS et al. Patterns of diseases and preventive measures among domestic hajjis from Central, Saudi Arabia [complete republication]. East Mediterr Health J. 2013;19 Suppl 2:S34-41. |
| Alfelali M. Facemask versus no facemask in preventing viral respiratory infections during Hajj: a cluster randomised open label trial (March 8, 2019). Available at SSRN: <https://ssrn.com/abstract=3349234> or [http://dx.doi.org/10.2139/ssrn.3349234](https://dx.doi.org/10.2139/ssrn.3349234) |
| Babak J et al. Impact of population mask wearing on Covid-19 post lockdown. medRxiv preprint doi: https://doi.org/10.1101/2020.04.13.20063529 |
| Bae S et al. Effectiveness of Surgical and Cotton Masks in Blocking SARS–CoV-2: A Controlled Comparison in 4 Patients. Ann Intern Med. 2020 Apr 6. doi: 10.7326/M20-1342. |
| Balaban V et al. Protective Practices and Respiratory Illness Among US Travelers to the 2009 Hajj. J Travel Med. 2012 May-Jun;19(3):163-8. doi: 10.1111/j.1708-8305.2012.00602.x. |
| Brienen NC et al. The effect of mask use on the spread of influenza during a pandemic. Risk Anal. 2010 Aug;30(8):1210-8. doi: 10.1111/j.1539-6924.2010.01428.x. |
| Chen SC, Liao CM. Modelling control measures to reduce the impact of pandemic influenza among schoolchildren. Epidemiol. Infect. (2008), 136, 1035–1045 doi:10.1017/S0950268807009284 |
| Choudhry AJ et al. Hajj-associated acute respiratory infection among hajjis from Riyadh. East Mediterr Health J. 2006 May-Jul;12(3-4):300-9. |
| Cio J et al. Influence of asymptomatic infections for the e_ectiveness of facemasks during pandemic influenza. Math Biosci Eng. 2019 May 6;16(5):3936-3946. doi: 10.3934/mbe.2019194. |
| D’Orazio M et al. How to restart? An agent-based simulation model towards the definition of strategies for COVID-19 “second phase” in public buildings. arXiv:2004.12927v1 [physics.soc-ph] |
| Davies A et al. Testing the Efficacy of Homemade Masks: Would They Protect in an Influenza Pandemic? Disaster Med Public Health Prep. 2013 Aug;7(4):413-8. doi: 10.1017/dmp.2013.43. |
| De Kai GPG et al. Universal Masking is Urgent in the COVID-19 Pandemic: SEIR and Agent Based Models, Empirical Validation, Policy Recommendations. arXiv:2004.13553v1 [physics.soc-ph] |
| Deris ZZ et al. The Prevalence of Acute Respiratory Symptoms and Role of Protective Measures Among Malaysian Hajj Pilgrims. J Travel Med. 2010 Mar-Apr;17(2):82-8. doi: 10.1111/j.1708-8305.2009.00384.x. |
| Eikenberry SE et al. To mask or not to mask: Modeling the potential for face mask use by the general public to curtail the COVID-19 pandemic. Infect Dis Model. 2020 Apr 21;5:293-308. doi: 10.1016/j.idm.2020.04.001. |
| Emamian MH et al. Respiratory Tract Infections and its Preventive Measures among Hajj Pilgrims, 2010: A Nested Case Control Study. Int J Prev Med. 2013 Sep;4(9):1030-5. |
| Guha S et al. Effectiveness of facemasks for pediatric populations against submicron-sized aerosols. Am J Infect Control. 2015 Aug;43(8):871-7. doi: 10.1016/j.ajic.2015.03.032. Epub 2015 May 26. |
| Kim CO et al. Is abdominal obesity associated with the 2009 influenza A (H1N1) pandemic in Korean school-aged children? Influenza Other Respir Viruses. 2012 Sep;6(5):313-7. doi: 10.1111/j.1750-2659.2011.00318.x |
| Lai AC et al. Effectiveness of facemasks to reduce exposure hazards for airborne infections among general populations. J R Soc Interface. 2012 May 7;9(70):938-48. doi: 10.1098/rsif.2011.0537. Epub 2011 Sep 21. |
| Lau JT et al. SARS Transmission, Risk Factors, and Prevention in Hong Kong. Emerg Infect Dis. 2004 Apr;10(4):587-92. |
| Li Y et al. Transmission of communicable respiratory infections and facemasks. J Multidiscip Healthc. 2008 May 1;1:17-27. |
| Makison Booth C et al. Effectiveness of surgical masks against influenza bioaerosols. J Hosp Infect. 2013 May;84(1):22-6. doi: 10.1016/j.jhin.2013.02.007. |
| Milton DK et al. Influenza Virus Aerosols in Human Exhaled Breath: Particle Size, Culturability, and Effect of Surgical Masks. PLoS Pathog. 2013 Mar;9(3):e1003205. doi: 10.1371/journal.ppat.1003205. |
| Mniszewski SM et al. Understanding the Impact of Face Mask Usage Through Epidemic Simulation of Large Social Networks. Chapter in Intelligent Systems Reference Library - October 2014 doi: 10.1007/978-3-642-39149-1_8 |
| Ngonghala CN et al. Mathematical assessment of the impact of non-pharmaceutical interventions on curtailing the 2019 novel Coronavirus. medRxiv preprint doi: <https://doi.org/10.1101/2020.04.15.20066480>. Now published in Mathematical Biosciences doi: 10.1016/j.mbs.2020.108364 |
| Regasamy S et al. Simple Respiratory Protection—Evaluation of the Filtration Performance of Cloth Masks and Common Fabric Materials Against 20–1000 nm Size Particles. Ann Occup Hyg. 2010 Oct;54(7):789-98. doi: 10.1093/annhyg/meq044 |
| Tian L et al. Calibrated Intervention and Containment of the COVID-19 Pandemic. arXiv:2003.07353v4 [q-bio.PE] |
| Tracht SM et al. Economic analysis of the use of facemasks during pandemic (H1N1) 2009. J Theor Biol. 2012 May 7;300:161-72. doi: 10.1016/j.jtbi.2012.01.032. Epub 2012 Jan 28. |
| Tracht SM et al. Mathematical modeling of the effectiveness of facemasks in reducing the spread of novel influenza A (H1N1). PLoS One. 2010 Feb 10;5(2):e9018. doi: 10.1371/journal.pone.0009018. |
| Uchida M et al. Effectiveness of vaccination and wearing masks on seasonal influenza in Matsumoto City, Japan, in the 2014/2015 season: An observational study among all elementary schoolchildren. Prev Med Rep. 2016 Dec 6;5:86-91. |
| van der Sande M et al. Professional and Home-Made Face Masks Reduce Exposure to Respiratory Infections among the General Population. PLoS One. 2008 Jul 9;3(7):e2618. doi: 10.1371/journal.pone.0002618. |
| Wu J et al. Risk Factors for SARS among Persons without Known Contact with SARS Patients, Beijing, China. Emerg Infect Dis. 2004 Feb;10(2):210-6. |
| Yan J et al. Modeling the Effectiveness of Respiratory Protective Devices in Reducing Influenza Outbreak. Risk Analysis, Vol. 39, No. 3, 2019 doi: 10.1111/risa.13181 |
| Zhang L et al. Protection by Face Masks against Influenza A(H1N1)pdm09 Virus on Trans-Pacific Passenger Aircraft, 2009. Emerg Infect Dis. 2013;19(9). doi: 10.3201/eid1909.121765. |

# Table S3 - Excluded studies

|  | Allison et al. (2010) Feasibility of elementary school children’s use of hand gel and facemasks during influenza season. Influenza and Other Respiratory Viruses 4(4), 223–229. | Not meeting inclusion criteria for type of intervention and population |
| --- | --- | --- |
|  | Amariles P, et al., How to link patients with suspicious COVID-19 to health system from the community pharmacies? A route proposal. Research in Social and Administrative Pharmacy, https://doi.org/10.1016/j.sapharm.2020.03.007. | Not meeting inclusion criteria |
|  | Anonymous. Summaries for patients. The effects of hand washing and facemasks on prevention of influenza infection. [Ann Intern Med.](https://www.ncbi.nlm.nih.gov/pubmed/19805764) 2009 Oct 6;151(7):I-18. Original report in “Facemasks and hand hygiene to prevent influenza transmission in households: a cluster randomized trial” DOI: 10.7326/0003-4819-151-7-200910060-00001 | Not meeting inclusion criteria for type of study and setting |
|  | Barasheed O et al. Pilot Randomised Controlled Trial to Test Effectiveness of Facemasks in Preventing Influenza-like Illness Transmission among Australian Hajj Pilgrims in 2011. Infectious Disorders – Drug Targets, 2014, 14, 110-116 | Not meeting inclusion criteria for population |
|  | bin-Reza et al. (2012) The use of masks and respirators to prevent transmission of influenza: a systematic review of the scientific evidence. Influenza and Other Respiratory Viruses 6(4), 257–267 | Not meeting inclusion criteria for type of study |
|  | Borkow G, Zhou SS, Page T, Gabbay J (2010) A Novel Anti-Influenza Copper Oxide Containing Respiratory Face Mask. PLoS ONE 5(6): e11295. doi:10.1371/journal.pone.0011295 | Not meeting inclusion criteria for intervention and outcome and setting |
|  | Chan DK et al. Preventing the spread of H1N1 influenza infection during a pandemic: autonomy-supportive advice versus controlling instruction. J Behav Med. 2015 Jun;38(3):416-26. doi: 10.1007/s10865-014-9616-z. Epub 2014 Dec 27. | Not meeting inclusion criteria for type of study |
|  | Chan KH, Yuen KY. COVID-19 epidemic: disentangling the re-emerging controversy about medical facemasks from an epidemiological perspective. nt J Epidemiol. 2020 Mar 31. pii: dyaa044. doi: 10.1093/ije/dyaa044 | Not meeting inclusion criteria for type of study |
|  | Cheng KK, Lam TH, Leung CC. Wearing face masks in the community during the COVID-19 pandemic: altruism and solidarity. Lancet. 2020 Apr 16. pii: S0140-6736(20)30918-1. doi: 10.1016/S0140-6736(20)30918-1 | Not meeting inclusion criteria for type of study |
|  | Chowell D et a. Sustainable social distancing through facemask use and testing during the Covid-19 pandemic. medRxiv preprint doi: <https://doi.org/10.1101/2020.04.01.20049981> | Effectiveness of facemask wearing alone cannot be obtained from PPE combined effectiveness results |
|  | Chung PK. The process by which perceived autonomy support predicts motivation, intention, and behavior for seasonal influenza prevention in Hong Kong older adults. BMC Public Health. 2017 Jul 28;18(1):65. doi: 10.1186/s12889-017-4608-x | Not meeting inclusion criteria for type of outcome |
|  | Cowling BJ et al. Face masks to prevent transmission of influenza virus: a systematic review. Epidemiol. Infect. (2010), 138, 449–456. doi:10.1017/S0950268809991658 | Not meeting inclusion criteria for type of study |
|  | Dargaville T, Spann K, Celina M. Opinion to address a potential personal protective equipment shortage in the global community during the COVID-19 outbreak. Polymer Degradation and Stability 176 (2020) 109162. https://doi.org/10.1016/j.polymdegradstab.2020.109162 | Not meeting inclusion criteria for type of study and outcome |
|  | Del Valle SY et al. Can we reduce the spread of influenza in schools with face masks? Am J Infect Control 2010;38:676-7 doi:10.1016/j.ajic.2010.03.012 | Not meeting inclusion criteria for type of study |
|  | Feng S et al. Rational use of face masks in the COVID-19 pandemic. Lancet Respir Med. 2020 May;8(5):434-436. doi: 10.1016/S2213-2600(20)30134-X. Epub 2020 Mar 20. | Not meeting inclusion criteria for Intervention |
|  | Gautret P et al. Protective Measures Against Acute Respiratory Symptoms in French Pilgrims Participating in the Hajj of 2009. Journal of Travel Medicine 2011; Volume 18 (Issue 1): 53–55 | No controls |
|  | Greenhalgh T et al. Face masks for the public during the covid-19 crisis. BMJ. 2020 Apr 9;369:m1435. doi: 10.1136/bmj.m1435. | Not meeting inclusion criteria for type of study |
|  | Hashim S. The prevalence and preventive measures of the respiratory illness among Malaysian pilgrims in 2013 hajj season. Journal of Travel Medicine, 2016, 1–7 doi: 10.1093/jtm/tav019 | No controls |
|  | Jolie R et al. Health Problems in Veterinary Students After Visiting a Commercial Swine Farm. Can J Vet Res 1998; 62: 44-48 | Not meeting inclusion criteria for setting |
|  | Larson EL et al. Impact of non-pharmaceutical interventions on URIs and influenza in crowded, urban households. Public Health Rep. 2010 Mar-Apr;125(2):178-91 | Not meeting inclusion criteria for setting |
|  | MacIntyre CR, Chughtai AA. Facemasks for the prevention of infection in healthcare and community settings. BMJ. 2015 Apr 9;350:h694. doi: 10.1136/bmj.h694. | Not meeting inclusion criteria for type of study and outcome |
|  | MacIntyre CR, Hasanain SJ. Community Universal Face Mask Use during the COVID 19 pandemic-from households to travelers and public spaces. J Travel Med. 2020 Apr 18. pii: taaa056. doi: 10.1093/jtm/taaa056. [Epub ahead of print] | Not meeting inclusion criteria for Intervention |
|  | Mahase E. Covid-19: What is the evidence for cloth masks? BMJ. 2020 Apr 7;369:m1422. doi: 10.1136/bmj.m1422 | Not meeting inclusion criteria for type of study |
|  | Mitka M. Face masks, respirators might help during pandemic flu outbreak. JAMA. 2007 Jun 6;297(21):2338. | Not meeting inclusion criteria for type of study |
|  | Mukerji S, MacIntyre CR, Newall AT. Review of economic evaluations of mask and respirator use for protection against respiratory infection transmission. BMC Infect Dis. 2015 Oct 13;15:413. doi: 10.1186/s12879-015-1167-6 | Not meeting inclusion criteria for type of study |
|  | Neilson S. The surgical mask is a bad fit for risk reduction. CMAJ. 2016 Apr 11. pii: cmaj.151236. [Epub ahead of print] | Not meeting inclusion criteria for type of study |
|  | Pan J et al. Effectiveness of control strategies for Coronavirus Disease 2019: a SEIR dynamic modeling study preprint doi: https://doi.org/10.1101/2020.02.19.20025387 | Not meeting inclusion criteria for type of outcome |
|  | Roberge R. Facemask use by children during infectious disease outbreaks. Biosecur Bioterror. 2011 Sep;9(3):225-31. doi: 10.1089/bsp.2011.0009. Epub 2011 Aug 15 | Study not meeting inclusion criteria for type of study |
|  | Saunders-Hastings P, Crispo JAG, Sikora L, Krewski D. Effectiveness of personal protective measures in reducing pandemic influenza transmission: A systematic review and meta-analysis. Epidemics. 2017 Sep;20:1-20. doi: 10.1016/j.epidem.2017.04.003. Epub 2017 Apr 30 | Not meeting inclusion criteria for type of study |
|  | Seto WH. Airborne transmission and precautions: Facts and myths. J Hosp Infect. 2015 Apr;89(4):225-8. doi: 10.1016/j.jhin.2014.11.005. Epub 2014 Dec 13 | Not meeting inclusion criteria for type of study |
|  | Sim SW, Moey KS, Tan NC. The use of facemasks to prevent respiratory infection: A literature review in the context of the Health Belief Model. Singapore Med J. 2014 Mar;55(3):160-7. | Not meeting inclusion criteria for type of study and outcome |
|  | Stern D et al. Rapid review of the use of community-wide surgical masks and acute respiratory infections. Salud Publica Mex. 2020 Apr 9. doi: 10.21149/11379. [Epub ahead of print] | Not meeting inclusion criteria for Intervention and type of study |
|  | Szarpak L et al. Cloth masks versus medical masks for COVID-19 protection. Cardiol J. 2020 Apr 14. doi: 10.5603/CJ.a2020.0054. [Epub ahead of print] | Not meeting inclusion criteria for type of study |
|  | Tahir MF et al. Seroprevalence and risk factors of avian influenza H9 virus amongpoultry professionals in Rawalpindi, Pakistan. Journal of Infection and Public Health 12 (2019) 482–485 | Not meeting inclusion criteria for setting |
|  | Taylor M et al. Public health measures during an anticipated influenza pandemic: Factors influencing willingness to comply. Risk Manag Healthc Policy. 2009;2:9-20. doi: 10.2147/RMHP.S4810. Epub 2009 Jan 29. | Not meeting inclusion criteria for outcome |
|  | Vogel L. Who should wear a face mask? Experts weigh in on Canada's COVID-19 response. CMAJ. 2020 Apr 20;192(16):E440-E441. doi: 10.1503/cmaj.1095863. | Not meeting inclusion criteria for type of study |
|  | Wada K et al. Wearing face masks in public during the influenza season may reflect other positive hygiene practices in Japan. BMC Public Health. 2012 Dec 10;12:1065. doi: 10.1186/1471-2458-12-1065. | Not meeting inclusion criteria for Intervention |
|  | Wang MW et al. Mask crisis during the COVID-19 outbreak. Eur Rev Med Pharmacol Sci. 2020 Mar;24(6):3397-3399. doi: 10.26355/eurrev_202003_20707. | Not meeting inclusion criteria for Intervention and type of study |
|  | Wang MW et al. The COVID-19 outbreak: issue of face masks. Infect Control Hosp Epidemiol. 2020 Apr 13:1-2. doi: 10.1017/ice.2020.129. [Epub ahead of print] | Not meeting inclusion criteria for type of study |
|  | Weiss MM et al. Disrupting the transmission of influenza a: face masks and ultraviolet light as control measures. Am J Public Health. 2007 Apr;97 Suppl 1:S32-7. Epub 2007 Apr 5 | Not meeting inclusion criteria for type of study and intervention |
|  | Venkateswaran J, Damani O Effectiveness of Testing, Tracing, Social Distancing and Hygiene in Tackling Covid-19 in India: A System Dynamics Model arXiv:2004.08859v1 [q-bio.PE] | Effectiveness of facemask wearing alone cannot be obtained from PPE combined effectiveness results |
|  | Wong VW, Cowling BJ, Aiello AE. Hand hygiene and risk of influenza virus infections in the community: a systematic review and meta-analysis. Epidemiol Infect. 2014 May;142(5):922-32. doi: 10.1017/S095026881400003X. Epub 2014 Jan 24 | Not meeting inclusion criteria for type of study and setting |
|  | Worby CJ, Chang HH. Face mask use in the general population and optimal resource allocation during the COVID-19 pandemic.. medRxiv preprint doi: <https://doi.org/10.1101/2020.04.04.20052696> | Not meeting inclusion criteria for type of outcome |
|  | Wu HL et al. Facemask shortage and the novel coronavirus disease (COVID-19) outbreak: Reflections on public health measures. EClinicalMedicine. 2020 Apr 3:100329. doi: 10.1016/j.eclinm.2020.100329 | Not meeting inclusion criteria for Outcome |
|  | Xiao J et al. Nonpharmaceutical Measures for Pandemic Influenza in Nonhealthcare Settings-Personal Protective and Environmental Measures. Emerg Infect Dis. 2020 May;26(5):967-975. doi: 10.3201/eid2605.190994. Epub 2020 May 17. | Not meeting inclusion criteria for type of study |
|  | Yuan EJ et al. Where to buy face masks? Survey of applications using Taiwan's open data in the time of COVID-19. J Chin Med Assoc. 2020 Apr 15. doi: 10.1097/JCMA.0000000000000325. | Not meeting inclusion criteria for intervention and outcome |
|  | Zhang CQ et al. Health Beliefs of Wearing Facemasks for Influenza A/H1N1 Prevention: A Qualitative Investigation of Hong Kong Older Adults. Asia Pac J Public Health. 2019 Apr;31(3):246-256. doi: 10.1177/1010539519844082. Epub 2019 Apr 21. | Not meeting inclusion criteria for type of study and outcome |

## **Appendix 3. Risk of bias of epidemiologic studies and unfeasibility of deterministic models**

#### Risk of bias of epidemiologic studies

Two reviewers independently assessed the below-mentioned domains of RoB. Any disagreement was resolved by consensus. For RCTs we used the Cochrane Risk of Bias tool including domains on random sequence generation, allocation concealment, blinding of participants and personnel, blinding of outcome assessors, incomplete outcome data, and selective reporting. Thus, in cluster-randomized trials, particular biases were considered: (i) recruitment bias; (ii) baseline imbalance; (iii) loss of clusters; (iv) incorrect analysis; and (v) comparability with individually randomized trials. Each item has been scored as “high,” “low,” or “unclear” RoB if no sufficient information is reported (Higgins 2011). For cohort studies, case control studies and cross sectional surveys we used the Newcastle-Ottawa Scale for controlling selection, comparability, exposure and outcomes. A study can be awarded a maximum of one star for each numbered item within the Selection and Exposure categories. A maximum of two stars can be given for Comparability (Wells 2010).

**
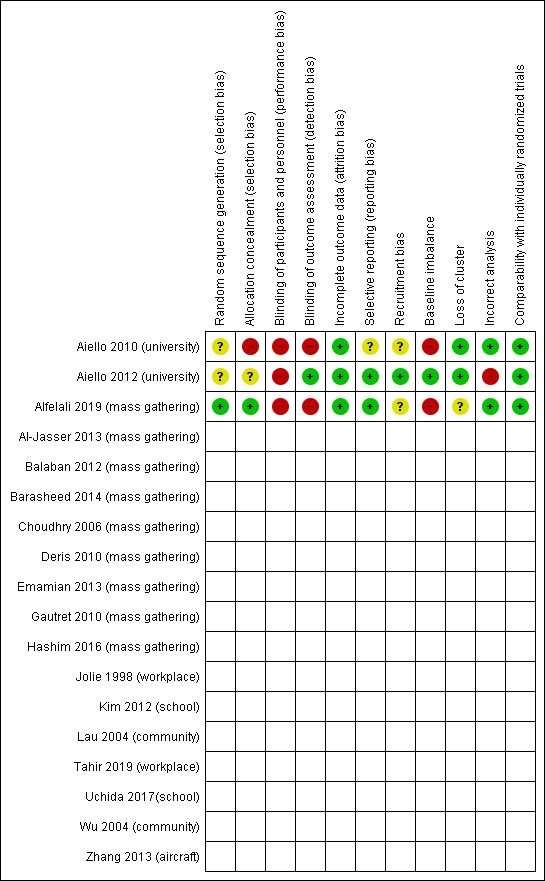
**

# Figure S1- Risk of bias in randomized controlled trials

# Table S4- Methodological quality of cohort studies

|  | **Selection** | | | | **Comparability** | **Outcome** | | |  |
| --- | --- | --- | --- | --- | --- | --- | --- | --- | --- |
| **Cohort study** | Representativeness of the exposed cohort | Selection of the non exposed cohort | Ascertainment of exposure | Demonstration that outcome of interest was not present at start of study | Comparability of cohorts on the basis of the design or analysis | Assessment of outcome | Was follow-up long enough for outcomes to occur | Adequacy of follow up of cohorts | \| **NOS** **quality score** (N. of stars) \| \| --- \| |
| Al-Jasser 2012 |  | * |  | * |  |  | * | * | poor |
| Balaban 2009 |  | * |  |  | * |  | * | * | poor |
| Choudhry 2006 |  | * |  | * | * |  | * | * | fair |

# Table S5- Methodological quality of case-control studies

|  | **Selection** | | | | **Comparability** | **Exposure** | | |  |
| --- | --- | --- | --- | --- | --- | --- | --- | --- | --- |
| **Case-control study** | Adequacy of case definition | Representativeness of the cases | Selection of controls | Definition of controls | Comparability of cases and controls on the basis of the design or analysis | Ascertainment of exposure | Same methods of ascertainment of cases and contros | Non-response rate | **NOS** **quality score** (N. of stars) |
| Emanian 2013 |  | * | * |  | * |  | * | * | fair |
| Lau 2004 |  | * | * |  | * |  | * |  | poor |
| Wu 2004 |  | * | * |  | * |  | * | * | fair |
| Zhang 2013 | * | * | * |  | * | * |  |  | fair |

# Table S6 - Methodological quality of cross-sectional studies

|  | **Selection** | | | | **Comparability** | **Outcome** | |  |
| --- | --- | --- | --- | --- | --- | --- | --- | --- |
| **Cross-sectional study** | Representativeness of sample | Sample size | Non respondents | Ascertainment of the exposure (risk factor) | Comparability of the subjects in different outcome groups on the basis of the design or analysis | Assessment of outcome | Statistical test | **NOS** **quality score** (N. of stars) |
| Deris 2010 |  | * |  | * | * | * | * | fair |
| Kim 2012 | * | * |  | * | * | * | * | fair |
| Uchida 2017 | * | * | * | * | ** | * | * | good |

#### Unfeasibility of deterministic models

Since we found only quantitative-deterministic models, (statistical) bias was not a suitable measure of model goodness. Moreover, available questionnaires in literature ([Jaime Caro et al., 2014](#_ENREF_39)) usually address the decision-making process as a whole and do not seem to have a model-centric perspective. Unlike statistical models and machine learning models, quantitative-deterministic models such as SIR-based and agent-based models are not fitted or trained on observed data to improve their performances. Available data are rather used to inform the model so that it can be adapted and calibrated on the task at hand. Thus, we had to use the checklist “QUAntitative-Deterministic models Risk of Infeasibility Assessment Checklist (QUADRIAC)” aiming at measuring the risk of “infeasibility”, namely the risk that a stakeholder might find the model neither reproducible nor trustworthy because of some too restrictive assumptions or some misleading steps that yield the solution, as well as the lack of credibility. The risk of unfeasibility was assessed by evaluating four selected criteria( model relevance, parameters uncertainty, reproducibility and credibility), which integrate the guidelines reported in Porgo et al. 2019 in order to better account for model uncertainty and reproducibility (Table S7) (Porgo 2019).

# Table S7 – QUAntitative-Deterministic models Risk of Infeasibility Assessment Checklist (QUADRIAC)

| Model relevance | *Were there some misleading or too restrictive assumptions?* |
| --- | --- |
|  | *Was the selected model suitable for the scope?* |
| Parameters uncertainty | *Was the input parameters chosen upon the best available evidences?* |
|  | *If calibrated, was the methodology well documented?* |
| Reproducibility | *Were the steps yielding the result proven or at least outlined?* |
|  | *Were there missing logical steps that could mislead the reader?* |
| Credibility | *Was the output of the model easily available and/or accessible?* |
|  | *Was the model validated against a real-life scenario?* |

Each criterion could score one point if the study met its conditions, zero otherwise. Then, an overall score was computed as the sum of such points and could range in 0-4. Based on this score, the risk of unfeasibility of the study was rated as low (0), medium (1-2) or high (3-4).The risk of unfeasibility is computed as the sum of all the binary values 0-1 assigned to each of the four selected criteria (Porgo 2019).

# Table S8 - Unfeasibility of deterministic models

|  | **A** | **B** | **C** | **D** | **Overall score** |
| --- | --- | --- | --- | --- | --- |
| Babak 2020 | 0 | 1 | 0 | 1 | medium |
| Brienen 2010 | 0 | 1 | 1 | 1 | high |
| Chen 2008 | 0 | 0 | 0 | 1 | medium |
| Cui 2019 | 0 | 0 | 0 | 1 | medium |
| De Kai 2020 | 0 | 0 | 0 | 1 | medium |
| D'Orazio 2020 | 0 | 0 | 1 | 1 | medium |
| Eikenberry 2020 | 0 | 0 | 0 | 0 | low |
| Mniszewski 2015 | 1 | 0 | 1 | 1 | high |
| Ngonghala 2020 | 0 | 0 | 0 | 0 | low |
| Tian 2020 | 0 | 0 | 1 | 0 | medium |
| Tracht 2010 | 0 | 0 | 0 | 1 | medium |
| Tracht 2012 | 0 | 0 | 0 | 1 | medium |
| Yan 2019 | 0 | 0 | 0 | 0 | low |

## **Appendix 4. Sensitivity analyses**

*
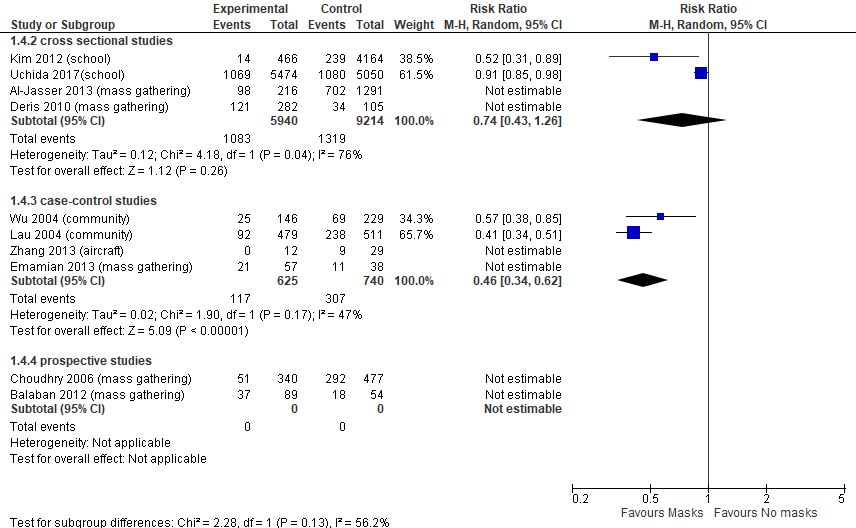
*

# Figure S2 - Sensitivity excluding aircraft and mass gathering studies.

## **Appendix 5. Additional analysis**

# Table S9. Outcomes Models

|  | **Epidemic level** | **Mask effecacy (%)** | **Decrease in susceptible (S)** | **Decrease in Infectively (I)** | **Population mask coverage (%)** | **R0** | **Respiratory infection rate (%)** | **Respiratory infection cases (as % of the initial state)** | **Respiratory infection cases (as the number of cumiulative case)** | | **Notes** |
| --- | --- | --- | --- | --- | --- | --- | --- | --- | --- | --- | --- |
| Brienen 2010 |  | - |  |  | 0 | 2.00 | 0.8 |  |  |  |  |
|  |  |  |  |  | 20 | 1.9 |  |  |  |  | extracted from graph |
|  |  | 30 |  |  | 80 |  | 0.6 |  |  |  |  |
|  |  |  |  |  | 100 | 1.4 |  |  |  |  |  |
|  |  | 70 |  |  | 20 |  | 0.7 |  |  |  | extracted from graph |
|  |  |  |  |  | 40 |  | 0.55 |  |  |  |  |
|  |  |  |  |  | 50 | 1.3 |  |  |  |  |  |
|  |  |  |  |  | 60 | 1.2 |  |  |  |  |  |
|  |  |  |  |  | 80 | 0.9 |  |  |  |  |  |
|  |  |  |  |  | 100 | 0.6 |  |  |  |  |  |
|  |  | 100 |  |  | 20 | 1.6 | 0.65 |  |  |  | extracted from graph |
|  |  |  |  |  | 40 | 1.2 | 0.3 |  |  |  |  |
|  |  |  |  |  | 50 | 1.0 | 0.0 |  |  |  |  |
|  |  |  |  |  | 80 | 0.4 |  |  |  |  |  |
|  |  |  |  |  | 100 | 0.0 |  |  |  |  |  |

# Table S10.Viral load reduction

| **Study** | **Groups** | **Sample** | **Particle Diameter** | **Total Colony-Forming Units** | **Median viral loads after coughs** | **Conclusion** |
| --- | --- | --- | --- | --- | --- | --- |
| Bae et al | -no mask  -homemade mask  -surgical mask | 4 patients |  |  | no mask: 2.56 log copies/mL  homemade mask: 1.85 log copies/mL  surgical mask: 2.42log copies/mL | This means a cotton mask blocked around 80% (reported as 0.7 log units or about a 5-fold decrease) of viral load on average, at eight inches away from a cough from a patient infected with COVID-19 |
| Davies et al | surgical mask  homemade mask | 21 volunteers | 2.1-3.3 μm  1.1-2.1 μm  0.65-1.1 μm | no mask:47  homemade mask:7  surgical mask: 5  no mask: 100 homemade mask: 16 surgical mask: 6  no mask: 21  homemade mask: 6 surgical mask: 3 |  | the surgical mask was 3 times more effective in reducing the number of microorganisms expelled than the homemade mask |
| Milton et al | surgical mask  no mask | 37 volunteers | particles < and > 5 mm | viral RNA:  wearing facemasks: 29 (78%)  not wearing facemasks: 35 (95%) | mean copy number in the fine particle fraction without a facemask was 110 (95% CI 45 to 260) and the facemasks produced a 2.8 fold  reduction in copy number (95% CI 1.5 to 5.2, p= 0.001).  Surgical masks produced a 3.4 (95% CI 1.8 to 6.3) fold reduction in viral copies in exhaled breath | The total viral copies detected by molecular methods were 8.8 times more numerous in fine (<5 mm) than in coarse (>5 mm) aerosol particles and that the fine particles from cases with the highest total number of viral RNA copies contained infectious virus. Surgical masks reduced the overall number of RNA copies by 3.4 fold. |

##

## **Appendix 6. PROSPERO protocol registration**

**PROTOCOL SYSTEMATIC REVIEW**

The effectiveness of wearing facemasks in the community for reducing the spread of COVID-19: a systematic review

**Review question**

- How effective is wearing or not wearing a mask in community setting?
- To investigate the effectiveness of wearing or not wearing a mask in real-life patients data (randomized controlled and observational studies)
- To predict the effectiveness of wearing or not wearing a mask (predictive model studies).
- Which masks is to be used in community setting?

**Searches**

We will search the following electronic databases: MEDLINE, EMBASE, SCISEARCH and The Cochrane Library to current date (up to 22 April 2020). We will search the following Grey databases: MedRxiv, Rxiv and bioRxiv databases. We will hand search the reference lists of the included papers. We will also review the studies included in any identified relevant systematic reviews.

**Types of study to be included**

No restrictions will be placed on study design. However, evidence will be prioritized by study design as follows: i) randomized controlled trials; ii) non-randomized comparative studies; iii) experimental studies. We will also review modelling studies.

**Condition or domain being studied**

Infectious respiratory disorders. Coronavirus disease 2019 (COVID-19) is a potentially severe acute respiratory infection caused by severe acute respiratory syndrome coronavirus 2 (SARS-CoV-2). The clinical presentation is that of a respiratory infection with a symptom severity ranging from a mild common cold-like illness, to a severe viral pneumonia leading to acute respiratory distress syndrome that is potentially fatal. Wearing mask in the community can play a key role in the prevention of infectious respiratory disorders.

**Participants/population**

Healthy people exposed to SARS-COV-2 infection [or SARS or MERS or H1N1 infection] in community setting. Population of health care workers will be excluded.

**Intervention(s), exposure(s)**

Any type of mask (ie. non-medical face mask (cloth, gauze, tissue etc), medical face mask and N95 respirators);

Any type of mask (ie. non-medical face mask (cloth, gauze, tissue etc), medical face mask and N95 respirators).

**Comparator(s)/control**

No mask

Any masks

**Context**

Setting: community setting for real life situations. Studies assessing the intervention in closed cluster such as households and health care setting will be excluded.

**Main outcome(s)**

To investigate:

- Mortality rate;
- Respiratory infection rate;
- Basic reproduction number (R0) of viral respiratory infections;
- Filtering capacity of masks;
- Viral load reduction.

in people exposed to SARS-COV-2 infection [or SARS or MERS or H1N1 or other influenza].

*** Measures of effect**

relative risks, odds ratios, risk difference, narrative summary (e.g. frequencies)

**Additional outcome(s)**

None

*** Measures of effect**

none

**Data extraction (selection and coding)**

Two independent reviewers will screen the titles and abstracts based on the eligibility criteria. The two reviewers should report the reasons for exclusion if documents are excluded. If there is a disagreement, it will be solved by discussion with a third reviewer.

A detailed data extraction form was developed prior to the systematic review being performed. Minimal data will be extracted addressing the following domains: study identifier; study design; setting; population characteristics; intervention and comparator characteristics; outcomes (quantitative if possible); study limitations or other important variables.

**Risk of bias (quality) assessment**

Two independent reviewers will perform risk of bias assessments. If there is a disagreement, it will be solved by discussion with a third reviewer.

We will use the Cochrane risk of bias tool for randomized controlled trials, the Newcastle Ottawa scale for non-randomized studies, the PROBAST (Prediction model Risk Of Bias Assessment Tool) for Prediction Model Studies or other checklist for deterministic mathematical models.

**Strategy for data synthesis**

We will synthesize data in both tabular and narrative formats. We anticipate our outcomes to be dichotomous, such as transmission, and therefore they will be analyzed as pooled risk ratios (RRs), if they are unadjusted estimates. If there are adjusted odds ratios from multivariable regression reported in the studies, then these will be pooled as adjusted odds ratios (aORs). These will be summarized using random effects meta-analysis using the DerSimonian and Laird random effects model, with heterogeneity calculated from the Mantel-Haenszel model. All summary measures will be reported with an accompanying 95% confidence interval. Data analyses will be performed using STATA 16.

GRADEpro GDT will be used to construct the summary of findings table. The analyses and reporting of the review will be done according to the PRISMA and MOOSE guidelines based on the study design included. Two independent reviewers will grade the certainty of the evidence using the GRADE approach. Evidence will be presented using GRADE Evidence Profiles developed in the GRADEpro (www.gradepro.org) software.

We will synthesize data in narrative formats for the filtering capacities of masks.

**Analysis of subgroups or subsets**

No subgroups.

**Contact details for further information**

Daniela Coclite ([daniela.coclite@iss.it](mailto:daniela.coclite@iss.it))

Organisational affiliation of the review

IRCCS Istituto Ortopedico Galeazzi, Unità di Epidemiologia Clinica

CNEC, ISS

**Review team members and their organisational affiliations**

Daniela Coclite

Antonello Napoletano

Greta Castellini

Silvia Gianola

Primiano Iannone

**Type and method of review**

Epidemiologic, Meta-analysis, Narrative synthesis, Systematic review

**Anticipated or actual start date**

22 April 2020

**Anticipated completion date**

25 May 2020

**Funding sources/sponsors**

Italian Ministry of Health (L2059)

**Conflicts of interest**

None

**Language**

English

**Country**

Italy

**Stage of review**

Review Ongoing

**Subject index terms status**

**Subject indexing assigned by CRD**

**Subject index terms**

COVID-19; Community; Humans; Infections; Masks; severe acute respiratory syndrome coronavirus 2; Influenza

**Date of registration in PROSPERO**

7 May 2020

**Date of publication of this version**

7 May 2020

**Details of any existing review of the same topic by the same authors**

Stage of review at time of this submission

## **Appendix 7. Reporting checklists**

# Table S11 - PRISMA checklist

| **Section/topic** | **#** | **Checklist item** | **Reported on page #** |
| --- | --- | --- | --- |
| **TITLE** | | |  |
| Title | 1 | Identify the report as a systematic review, meta-analysis, or both. | Page1 |
| **ABSTRACT** | | |  |
| Structured summary | 2 | Provide a structured summary including, as applicable: background; objectives; data sources; study eligibility criteria, participants, and interventions; study appraisal and synthesis methods; results; limitations; conclusions and implications of key findings; systematic review registration number. | Page 2 |
| **INTRODUCTION** | | |  |
| Rationale | 3 | Describe the rationale for the review in the context of what is already known. | Page 5 |
| Objectives | 4 | Provide an explicit statement of questions being addressed with reference to participants, interventions, comparisons, outcomes, and study design (PICOS). | Page 6-7 |
| **METHODS** | | |  |
| Protocol and registration | 5 | Indicate if a review protocol exists, if and where it can be accessed (e.g., Web address), and, if available, provide registration information including registration number. | Page 2 and page 6 |
| Eligibility criteria | 6 | Specify study characteristics (e.g., PICOS, length of follow-up) and report characteristics (e.g., years considered, language, publication status) used as criteria for eligibility, giving rationale. | Page 6-7 |
| Information sources | 7 | Describe all information sources (e.g., databases with dates of coverage, contact with study authors to identify additional studies) in the search and date last searched. | Page 6 |
| Search | 8 | Present full electronic search strategy for at least one database, including any limits used, such that it could be repeated. | Page 6 |
| Study selection | 9 | State the process for selecting studies (i.e., screening, eligibility, included in systematic review, and, if applicable, included in the meta-analysis). | Page 7 |
| Data collection process | 10 | Describe method of data extraction from reports (e.g., piloted forms, independently, in duplicate) and any processes for obtaining and confirming data from investigators. | Page 7-8 |
| Data items | 11 | List and define all variables for which data were sought (e.g., PICOS, funding sources) and any assumptions and simplifications made. | Page 7-8 |
| Risk of bias in individual studies | 12 | Describe methods used for assessing risk of bias of individual studies (including specification of whether this was done at the study or outcome level), and how this information is to be used in any data synthesis. | Page 8-9 |
| Summary measures | 13 | State the principal summary measures (e.g., risk ratio, difference in means). | Page 8 |
| Synthesis of results | 14 | Describe the methods of handling data and combining results of studies, if done, including measures of consistency (e.g., I^2^) for each meta-analysis. | Page 8 |
| Risk of bias across studies | 15 | Specify any assessment of risk of bias that may affect the cumulative evidence (e.g., publication bias, selective reporting within studies). | NA |
| Additional analyses | 16 | Describe methods of additional analyses (e.g., sensitivity or subgroup analyses, meta-regression), if done, indicating which were pre-specified. | Page 8 |
| **RESULTS** | | |  |
| Study selection | 17 | Give numbers of studies screened, assessed for eligibility, and included in the review, with reasons for exclusions at each stage, ideally with a flow diagram. | Page 9-10 |
| Study characteristics | 18 | For each study, present characteristics for which data were extracted (e.g., study size, PICOS, follow-up period) and provide the citations. | Pages 11-13 |
| Risk of bias within studies | 19 | Present data on risk of bias of each study and, if available, any outcome level assessment (see item 12). | Page 14 |
| Results of individual studies | 20 | For all outcomes considered (benefits or harms), present, for each study: (a) simple summary data for each intervention group (b) effect estimates and confidence intervals, ideally with a forest plot. | From Page 14 |
| Synthesis of results | 21 | Present results of each meta-analysis done, including confidence intervals and measures of consistency. | Pages 15-16 |
| Risk of bias across studies | 22 | Present results of any assessment of risk of bias across studies (see Item 15). | NA |
| Additional analysis | 23 | Give results of additional analyses, if done (e.g., sensitivity or subgroup analyses, meta-regression [see Item 16]). | Page 16 |
| **DISCUSSION** | | |  |
| Summary of evidence | 24 | Summarize the main findings including the strength of evidence for each main outcome; consider their relevance to key groups (e.g., healthcare providers, users, and policy makers). | Pages 20-22 |
| Limitations | 25 | Discuss limitations at study and outcome level (e.g., risk of bias), and at review-level (e.g., incomplete retrieval of identified research, reporting bias). | Page 25 |
| Conclusions | 26 | Provide a general interpretation of the results in the context of other evidence, and implications for future research. | Page 26 |
| **FUNDING** | | |  |
| Funding | 27 | Describe sources of funding for the systematic review and other support (e.g., supply of data); role of funders for the systematic review. | Page 28 |

*From:* Moher D, Liberati A, Tetzlaff J, Altman DG, The PRISMA Group (2009). Preferred Reporting Items for Systematic Reviews and Meta-Analyses: The PRISMA Statement. PLoS Med 6(7): e1000097. doi:10.1371/journal.pmed1000097

For more information, visit: **www.prisma-statement.org**.

# Table S12- MOOSE checklists

| **Item** **No** | **Recommendation** | **Reported** **on** **Page** **No** |
| --- | --- | --- |
| Reporting of background should include | | |
| 1 | Problem definition | Page 5-6 |
| 2 | Hypothesis statement | Page 5-6 |
| 3 | Description of study outcome(s) | Page 8 |
| 4 | Type of exposure or intervention used | Page 6-7 |
| 5 | Type of study designs used | Page 6-7 |
| 6 | Study population | Page 6-7 |
| Reporting of search strategy should include | | |
| 7 | Qualifications of searchers (eg, librarians and investigators) | Page 6 |
| 8 | Search strategy, including time period included in the synthesis and key words | Page 6 |
| 9 | Effort to include all available studies, including contact with authors | Page 6 |
| 10 | Databases and registries searched | Page 6 |
| 11 | Search software used, name and version, including special features used (eg, explosion) | Page 6 |
| 12 | Use of hand searching (eg, reference lists of obtained articles) | Page 6 |
| 13 | List of citations located and those excluded, including justification | Page 11 |
| 14 | Method of addressing articles published in languages other than English | Page 6 |
| 15 | Method of handling abstracts and unpublished studies | Page 6 |
| 16 | Description of any contact with authors | Page 6 |
| Reporting of methods should include | | |
| 17 | Description of relevance or appropriateness of studies assembled for assessing the hypothesis to be tested | Page 8-9 |
| 18 | Rationale for the selection and coding of data (eg, sound clinical principles or convenience) | Page 9 |
| 19 | Documentation of how data were classified and coded (eg, multiple raters, blinding and interrater reliability) | Page 9 |
| 20 | Assessment of confounding (eg, comparability of cases and controls in studies where appropriate) | NA |
| 21 | Assessment of study quality, including blinding of quality assessors, stratification or regression on possible predictors of study results | Page 9 |
| 22 | Assessment of heterogeneity | Page 8 |
| 23 | Description of statistical methods (eg, complete description of fixed or random effects models, justification of whether the chosen models account for predictors of study results, dose-response models, or cumulative meta-analysis) in sufficient detail to be replicated | Page 8 |
| 24 | Provision of appropriate tables and graphics | Page 8 |
| Reporting of results should include | | |
| 25 | Graphic summarizing individual study estimates and overall estimate | Pages 11-13 |
| 26 | Table giving descriptive information for each study included | Pages 11-13 |
| 27 | Results of sensitivity testing (eg, subgroup analysis) | Page 16 |
| 28 | Indication of statistical uncertainty of findings | Pages 20-22 |
| Reporting of discussion should include | | |
| 29 | Quantitative assessment of bias (eg, publication bias) | NA |
| 30 | Justification for exclusion (eg, exclusion of non-English language citations) | Page 10 |
| 31 | Assessment of quality of included studies | Page 14 |
| Reporting of conclusions should include | | |
| 32 | Consideration of alternative explanations for observed results | Pages 23-26 |
| 33 | Generalization of the conclusions (ie, appropriate for the data presented and within the domain of the literature review) | Page 23-26 |
| 34 | Guidelines for future research | Page 26 |
| 35 | Disclosure of funding source | Page 28 |

*From*: Stroup DF, Berlin JA, Morton SC, et al, for the Meta-analysis Of Observational Studies in Epidemiology (MOOSE) Group. Meta-analysis of Observational Studies in Epidemiology. A Proposal for Reporting. *JAMA*. 2000;283(15):2008-2012. doi: 10.1001/jama.283.15.2008.

Transcribed from the original paper within the NEUROSURGERY® Editorial Office, Atlanta, GA, United Sates. August 2012.

## **Appendix 8. References of Supplements**

Moher D, Liberati A, Tetzlaff J, Altman DG, The PRISMA Group (2009). Preferred Reporting Items for Systematic Reviews and Meta-Analyses: The PRISMA Statement. PLoS Med 6(7): e1000097. doi:10.1371/journal.pmed1000097

Higgins JPT, Green S (editors). Cochrane Handbook for Systematic Reviews of Interventions Version 5.1.0 [updated March 2011]. In: The Cochrane Collaboration. Available from www.cochrane-handbook.org. 2011.

Porgo, TV et al. The use of mathematical modeling studies for evidence synthesis and guideline development: A glossary. Res Synth Methods. 2019 Mar;10(1):125-133. doi: 10.1002/jrsm.1333. Epub 2019 Jan 8.

Stroup DF, Berlin JA, Morton SC, et al, for the Meta-analysis Of Observational Studies in Epidemiology (MOOSE) Group. Meta-analysis of Observational Studies in Epidemiology. A Proposal for Reporting. JAMA. 2000;283(15):2008-2012. doi: 10.1001/jama.283.15.2008.

Wells GA, Shea B, O'Connell D, et al. The Newcastle-Ottawa Scale (NOS) for assessing the quality of non randomised studies in meta-analyses. http://www.ohri.ca/programs/clinical_epidemiology/oxford.htm, 2010[last accessed 010/03/2015]
